# A Temporal–Section–Lane–Class Network for Freeway Lane-Level Mixed Traffic Flow Prediction

**DOI:** 10.3390/s26196287

**Published:** 2026-10-04

**Authors:** Heyao Gao, Jing Zhang, Hongfei Jia, Feiyan Li, Lin Hou, Chunhou Yang, Xin Li

**Affiliations:** 1 School of Transportation Science and Engineering, Jilin Jianzhu University, Changchun 130118, China; gaoheyao@jlju.edu.cn (H.G.); 19963827744@163.com (J.Z.); lifeiyan@jlju.edu.cn (F.L.); houlin@jlju.edu.cn (L.H.); yangchunhou@student.jlju.edu.cn (C.Y.); 13578576378@163.com (X.L.); 2 School of Transportation, Jilin University, Changchun 130012, China

**Keywords:** traffic flow prediction, mixed traffic, connected and automated vehicles, graph attention network

## Abstract

In vehicle-infrastructure cooperative systems, mixed freeway traffic comprising human-driven vehicles (HVs) and connected and automated vehicles (CAVs) presents new challenges for fine-grained traffic state monitoring and control. Lane changes and queue propagation, together with differences in HV and CAV driving behavior, produce traffic states that vary across space and vehicle type. We formulate lane-level mixed-traffic flow prediction as a joint forecasting task over the section, lane, and vehicle-class dimensions and develop a Temporal–Section–Lane–Class Network (TSLCN). A shared gated causal temporal convolutional network captures common temporal dynamics, while class-specific adapters preserve class-dependent characteristics. A section–lane–class (SLC) interaction module models longitudinal associations across monitoring sections, lateral interactions between adjacent lanes, and local HV–CAV dependencies. These representations yield class-specific forecasts for every lane at each monitoring section. Experiments combined observed lane flow data with mixed traffic scenarios simulated in Simulation of Urban MObility (SUMO) using demand obtained from field data. At 30% CAV penetration, TSLCN achieved the lowest aggregate MAE, RMSE, and WAPE and the lowest mean error in 68 of 72 comparisons. Its MAE reductions over the strongest baseline were statistically significant at all forecast horizons. Ablation analysis showed that each principal component contributed to prediction accuracy. TSLCN also achieved the lowest overall WAPE at CAV penetration rates from 0% to 100% and the lowest aggregate errors on an alternative freeway, with only modest sensitivity to key SUMO parameters. Overall, TSLCN provides reliable forecasts by lane and vehicle class, with potential applications in fine-grained freeway management.

## 1. Introduction

Short-term traffic flow prediction is an important component of intelligent transportation systems, providing anticipatory information for congestion management and traffic control [1]. As vehicle-infrastructure cooperation develops, human-driven vehicles (HVs) and connected and automated vehicles (CAVs) are expected to coexist for an extended transition period. The two vehicle classes differ in longitudinal car-following and lateral lane-changing behavior. Their relative proportions and spatial distributions can therefore affect traffic flow stability and roadway capacity [2]. Vehicle-infrastructure cooperative systems support increasingly fine-grained measures, including lane-level variable speed limits and dynamic lane assignment [3]. These capabilities motivate traffic forecasts that resolve flow conditions by lane and vehicle class.

Existing freeway traffic forecasting methods commonly operate at the cross-section or road-segment level. Such aggregation supports network-level traffic assessment but masks how flow is distributed across lanes and vehicle classes. Two time intervals with similar total cross-section flows may nevertheless exhibit different lane loads and vehicle compositions, leading to distinct local operating conditions and control requirements. Near entrance and exit ramps, merging, diverging, and lane-changing maneuvers generate pronounced short-term fluctuations in the outer lanes. Aggregation across a cross-section can obscure these local variations, reducing the information available for fine-grained traffic control. Lane-level prediction has been investigated to resolve traffic states at a finer spatial scale [4]. Extending this resolution to mixed traffic calls for representations that distinguish flows by vehicle class and preserve lane-level spatial relationships.

Recent deep-learning models have improved the representation of spatiotemporal dependencies across multiple road-network scales [5]. Lane-level studies have represented spatial relationships among lane segments [6], while mixed-traffic forecasting has incorporated HV and CAV flows from different sections and lanes [7]. Vehicle movements and queue propagation link traffic conditions at successive monitoring sections, while lane changes redistribute flow among neighboring lanes. At a given section, HV and CAV flows in the same lane are subject to common demand and capacity constraints but may respond differently because of their driving behavior. Joint prediction along the corridor should therefore account for traffic propagation between sections, flow redistribution among neighboring lanes, and local interactions between HV and CAV flows.

Accordingly, we propose a Temporal–Section–Lane–Class Network (TSLCN) for lane-level mixed traffic flow prediction on freeways. A shared gated causal temporal convolutional network learns temporal patterns common to historical HV and CAV flows. Class-specific adapters then refine these shared representations to account for class-dependent temporal characteristics. The resulting temporal representations are passed to a section–lane–class (SLC) interaction module, which applies three relation-specific components sequentially. Section graph attention captures longitudinal associations across monitoring sections, and adjacency-constrained lane graph attention models lateral interactions between adjacent lanes. A vehicle-class interaction gate then regulates information exchange between HV and CAV representations within each section–lane unit. The resulting features are decoded into class-specific flow forecasts for every lane at each monitoring section.

The contributions of this study are summarized as follows:

- Lane-level mixed traffic flow prediction is formulated as a joint task over monitoring sections, lanes, and vehicle classes. TSLCN forecasts all section–lane–class targets within a single model, combining a shared temporal encoder for common patterns with separate HV and CAV adapters for their distinct dynamics.
- The SLC interaction module models longitudinal section associations, lateral lane interactions, and local HV–CAV dependencies separately. Section attention incorporates signed relative section positions, lane attention restricts direct cross-lane exchange to adjacent lanes, and class-specific gates regulate information received from the other vehicle class within each section–lane unit.
- Fine-grained comparisons show broadly consistent gains across vehicle classes, lanes, and forecast horizons, while ablation analyses identify complementary roles for temporal encoding, class-specific adaptation, and SLC interactions. Across the robustness tests, TSLCN ranks first at all seven CAV penetration levels from 0% to 100% and under most selected SUMO parameter settings, recording the lowest errors on the second freeway for both field-observed and simulated lane flows.

The remainder of this paper is organized into Related Work (Section 2), Problem Formulation (Section 3), Methodology (Section 4), Experiments and Results (Section 5), and Conclusions (Section 6).

## 2. Related Work

### 2.1. Traffic Flow Prediction Methods

Traffic flow prediction estimates future traffic states from historical observations. Relevant methods have evolved from statistical time-series and conventional machine-learning models to deep-learning architectures for representing complex traffic dynamics [8]. Early studies relied primarily on historical averages, autoregressive models, and autoregressive integrated moving average (ARIMA) models. Williams and Hoel introduced a seasonal ARIMA model to capture periodic variations in traffic flow [9]. Statistical models represent trends and periodicity with relatively few parameters, but their assumptions may be inadequate under abrupt demand changes and spatially propagated disturbances. Machine-learning methods such as support vector regression (SVR), random forest (RF), and k-nearest neighbors (KNN) were subsequently used to learn nonlinear mappings between observed traffic data and prediction targets [10]. Their performance depends on input construction, feature engineering, and hyperparameter selection, with temporal information typically introduced through manually specified lagged inputs.

Deep learning subsequently enabled richer representations of traffic patterns. Lv et al. used stacked autoencoders to learn hierarchical traffic flow features [11], whereas Ma et al. employed a long short-term memory (LSTM) network to model dependencies among historical states [12]. Zhao et al. developed a temporal convolutional network (TCN)-based approach for short-term traffic flow prediction. Causal and dilated convolutions were used to extract traffic variations at multiple time scales, and the model was validated on real-world traffic data [13]. Beyond traffic flow forecasting, Li et al. developed TCN-SA for vehicle trajectory prediction, combining temporal convolution with social convolutional pooling and self-attention to represent local and global interactions between vehicles [14].

### 2.2. Spatiotemporal Traffic Prediction

Traffic flow prediction depends on temporal evolution and conditions at neighboring or functionally related road locations, making joint spatiotemporal modeling essential. Ma et al. arranged traffic states across locations and time steps into regular matrices and used a convolutional neural network (CNN) to extract local spatiotemporal features [15]. Such methods assume fixed grid-based neighborhoods, whereas road networks exhibit directed and irregular connectivity. Graph neural networks (GNNs) instead represent monitoring locations as nodes and their connections as edges, providing a topology-aware basis for spatial traffic modeling [16].

Early GNN-based forecasting models primarily relied on predefined road-network topology. Yu et al. proposed the spatiotemporal graph convolutional network (STGCN), combining graph and temporal convolutions to learn spatial and temporal features, respectively [17]. Li et al. formulated traffic propagation as diffusion on a directed graph in the diffusion convolutional recurrent neural network (DCRNN), coupling diffusion convolution with recurrent units [18]. Guo et al. introduced spatial and temporal attention into the attention-based spatial-temporal graph convolutional network (ASTGCN) to identify influential nodes and time steps [19]. Guo and Yuan combined graph attention with temporal convolution in GATCN to capture spatial and temporal dependencies in short-term traffic speed forecasting [20]. Later models relaxed the assumption of a fixed topology. Graph WaveNet, the adaptive graph convolutional recurrent network (AGCRN), and the graph multi-attention network (GMAN) modeled latent dependencies using adaptive adjacency matrices, node-specific parameters, and spatiotemporal attention, respectively [21,22,23]. More recent studies incorporated traffic propagation delays and differentiated node roles within graph-based forecasting frameworks [24,25].

Collectively, these methods advanced graph-based traffic forecasting from fixed topology toward adaptive, dynamic, and node-specific spatial representations. However, nodes are still commonly defined at the detector, road-segment, or regional level [26]. Applying these approaches to lane-level mixed traffic forecasting involves finer-grained representations of traffic entities and spatial relationships.

### 2.3. Lane-Level and Mixed Traffic Flow Prediction

Cross-section-level forecasts aggregate multiple lanes at the same location, obscuring differences in their functional roles and local operating conditions. Lane-level prediction provides more detailed traffic information for applications such as lane control and ramp management. Existing methods differ in prediction scope and spatial representation. Gu et al. proposed the fusion deep learning (FDL) model, which uses entropy-based grey relational analysis to select lane sections associated with the target and a network combining LSTM and gated recurrent unit (GRU) layers to forecast the target speed [27]. Shen et al. developed the spatial-temporal attention-based fusion network (ST-AFN) for urban roads, which combines a speed-processing network with spatial attention over candidate lanes and a temporal-attention decoder to forecast flow at a selected target lane [28]. Lu et al. proposed the mixed deep learning (MDL) model, which selects input variables using the maximal information coefficient (MIC), arranges speed and flow observations from multiple sections and lanes as spatial arrays, and jointly forecasts lane speeds at the modeled section–lane units through convolutional LSTM (ConvLSTM) and convolutional layers [29]. Subsequent graph-based models represented lanes or lane segments as nodes and modeled their relationships through heterogeneous data and dynamic graphs [30], data-driven adjacency and gated graph recurrence [31], or graph convolutional recurrent networks [32]. Li et al. developed GraphMLP within a unified graph-based formulation of lane-level traffic forecasting. The model learns spatial features from a dynamic lane-segment graph, combines them with an independent temporal multilayer perceptron (MLP), and produces multistep forecasts of a single aggregate traffic variable, such as speed or flow, for all lane-segment nodes [6]. Across these formulations, each lane-level target is represented by a single speed or flow variable for the combined traffic stream.

Several mixed-traffic studies have examined how CAV penetration and class-specific driving behavior affect freeway operation. Yao et al. used separate car-following models for HVs and CAVs to analyze traffic stability and fundamental diagrams at different CAV penetration rates [33]. Pan et al. developed a multiclass multilane cell transmission model that represents class-specific headways, lane-changing rules, and lane-level flow propagation under several penetration scenarios [34]. Zhang and Yang incorporated mixed-traffic lane-changing behavior into a discrete macroscopic flow model [2]. These studies characterize traffic stability, capacity, and flow propagation under different assumptions about vehicle behavior and CAV penetration. Vehicle classes have also been considered in lane-level forecasting. Zheng et al. proposed the spatiotemporal attention-based encoder–decoder (STA-ED) model for lane-level flows from three vehicle classes (small and medium-sized passenger cars, small and medium-sized trucks, and large buses and trucks). Its spatial attention weights lane–class sequences according to their relevance to a selected target, while temporal attention within an LSTM encoder–decoder supports multistep forecasting [35]. Gao et al. proposed the hybrid DATCN model to predict the HV or CAV flow at a specified section and lane. For each target, dynamic time warping (DTW) selects related input series, feature attention weights their contributions, and a TCN produces the forecast [7]. Table 1 compares these methods in terms of their forecast targets, vehicle representations, modeling of spatial and cross-series relationships, and prediction scope.

Taken together, existing lane-level methods forecast aggregate traffic at either a selected lane or multiple lane locations, whereas class-specific methods focus on an individual prediction target. Among the studies reviewed above, class-specific lane flows over successive monitoring sections have not been formulated as a joint prediction problem. At the corridor scale, these flows evolve jointly because vehicle movements and queue propagation connect successive sections, lane changes redistribute traffic among neighboring lanes, and differences in HV and CAV driving behavior affect flow evolution within each lane. This setting motivates corridor-level forecasting that preserves both lane and vehicle-class information and accounts for the dependencies among the resulting flow series.

## 3. Preliminary Analysis and Problem Formulation

### 3.1. Lane-Level Mixed Traffic Flow Representation

Consider a freeway segment with *S* monitoring sections, which are ordered along the direction of travel and each cover *L* lanes. HV and CAV flows are recorded separately at fixed time intervals. Let C∈{HV,CAV} denote the set of vehicle classes, and let *q_t_*_,*s*,*l*,*c*_ denote the observed flow of class c∈C at time step *t*, monitoring section *s* and lane *l*. Arranging all observations at time step *t* along the section, lane, and vehicle-class dimensions yields the lane-level mixed traffic state tensor (***Q****_t_*):

***Q****_t_* = [*q_t_*_,*s*,*l*,*c*_] ∈ ℝ*^S^*^×*L*×*C*^ (1)

where *s* = 1, …, *S*, *l* = 1, …, *L*, and *C* is the number of vehicle classes. In this study, C=|C|=2, corresponding to HVs and CAVs. Aggregation of ***Q****_t_* over vehicle classes yields the class-aggregated lane flow, and subsequent aggregation over lanes yields the cross-section flow. These quantities are expressed as follows:

$$q_{t,s,l}^{\mathrm{lane}}=\sum_{c\in C}q_{t,s,l,c},\;q_{t,s}^{\mathrm{section}}=\sum_{l=1}^{L}q_{t,s,l}^{\mathrm{lane}}$$

The first aggregation retains the lane distribution while combining HV and CAV flows within each lane; the second also combines flows across lanes. The tensor ***Q****_t_* retains both the lane distribution and vehicle-class composition.

**Figure 1:**
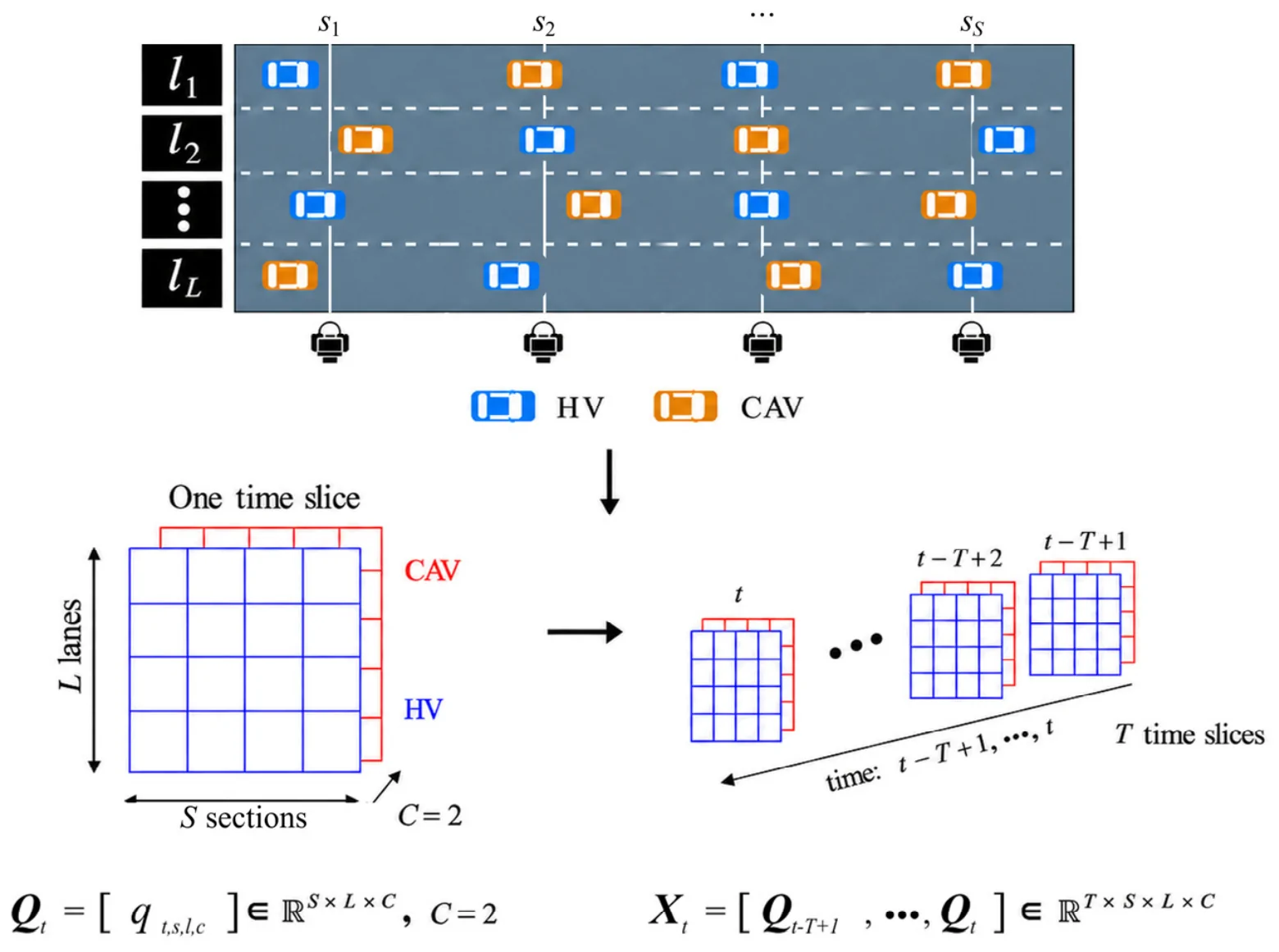
Representation of lane-level mixed traffic flow.

As illustrated in Figure 1, each section–lane unit (a specific lane at a given monitoring cross-section) is associated with an HV flow observation and a CAV flow observation at each time step. The observations from all section–lane units constitute the traffic state tensor ***Q****_t_*. Stacking the state tensors from *T* consecutive time steps in chronological order gives the historical observation sequence (***X****_t_*):

***X****_t_* = [***Q****_t_*_−*T*+1_, …, ***Q****_t_*] ∈ ℝ*^T^*^×*S*×*L*×*C*^ (2)

where *T* denotes the length of the historical observation window.

### 3.2. Association Characteristics of Lane-Level Mixed Traffic Flow

**Figure 2:**
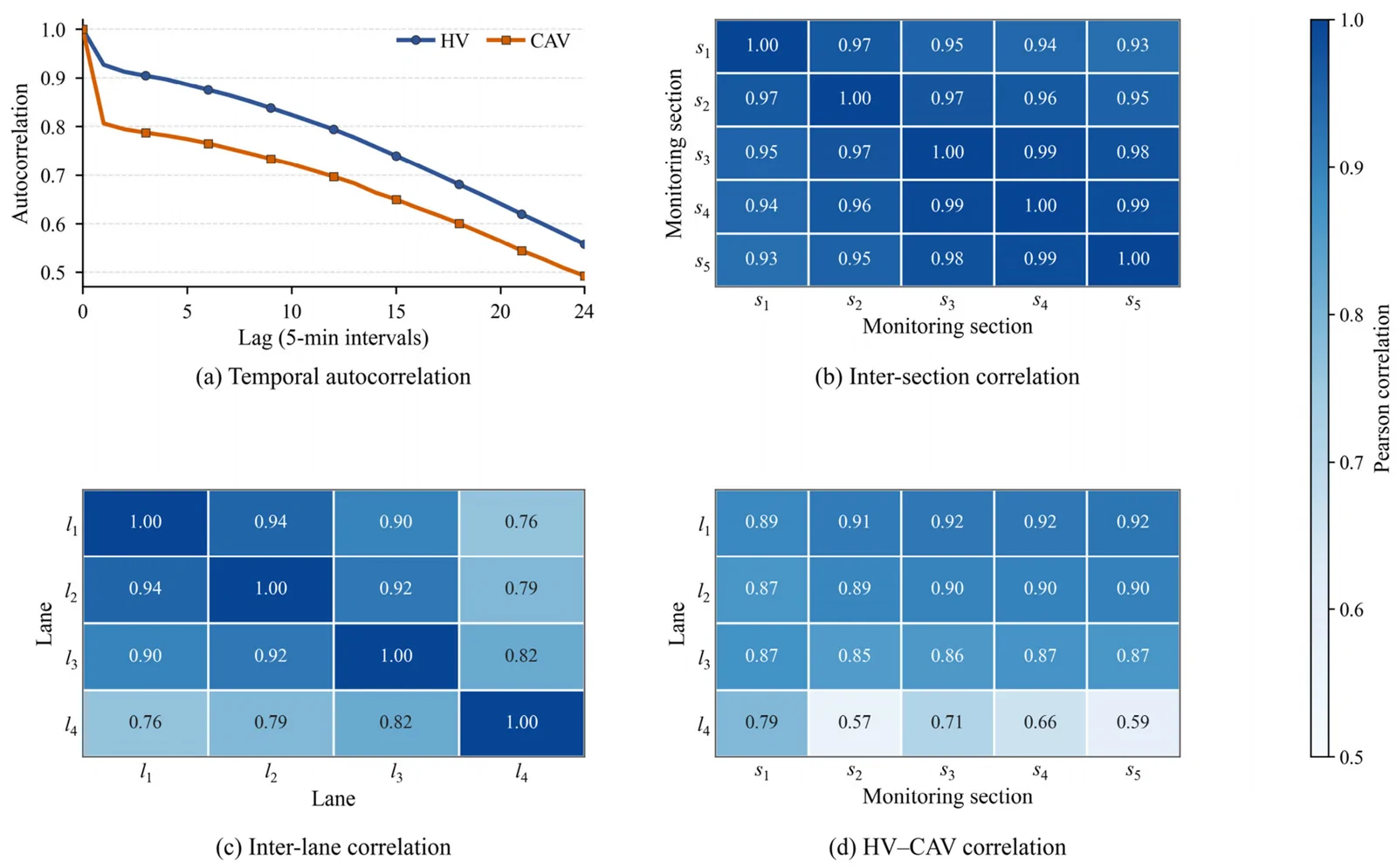
Correlation patterns in the training partition of the 30% CAV penetration scenario.

Lane-level mixed traffic flow can exhibit temporal, inter-section, inter-lane, and cross-class associations. Figure 2 provides an empirical illustration of these patterns using raw flows from the chronological training partition of the 30% CAV penetration scenario. Temporal autocorrelations were calculated separately for HV and CAV flows and averaged across the 20 section–lane units. Inter-section correlations were averaged across four lanes and two vehicle classes, whereas inter-lane correlations were averaged across five monitoring sections and two classes. Fisher’s *z*-transformation was applied before averaging these coefficients. HV–CAV correlations were calculated directly within each section–lane unit.

Temporal persistence arises because traffic demand and operating regimes generally change gradually, while queues require time to form and dissipate. Successive aggregation intervals therefore tend to exhibit similar traffic states. Longitudinal coupling can arise from vehicle conservation, finite travel times, and the propagation of queues and disturbances. Common demand patterns may also induce contemporaneous variation across monitoring sections. Inter-lane associations can result from direct flow redistribution through lane changes and from the shared response of lanes to demand, capacity constraints, and ramp activity. Figure 2a–c illustrate these temporal and spatial patterns in the analyzed scenario.

Within each section–lane unit, HV and CAV flows are jointly influenced by local demand and available roadway capacity. The two classes may also interact through car-following and lane-changing maneuvers. Differences in response time, headway, and lane use contribute to location-dependent cross-class variation. Figure 2d shows positive HV–CAV correlations, with comparatively weaker associations in the outer lane of the analyzed corridor. Together, these mechanisms characterize lane-level mixed traffic as a multidimensional process. The strength and spatial extent of the resulting associations can vary with traffic composition and roadway conditions.

### 3.3. Lane-Level Mixed Traffic Flow Prediction Task

Given the historical observation sequence (***X****_t_*) defined in Section 3.1, the objective of lane-level mixed traffic flow prediction is to use the traffic states from the preceding *T* time steps to predict the flows of each vehicle class in every lane at each monitoring section over the next *H* time steps. Arranging the observed future traffic states over the forecast horizon in chronological order gives the prediction target (***Y****_t_*):

***Y****_t_* = [***Q****_t_*_+1_, …, ***Q****_t_*_+*H*_] ∈ ℝ*^H^*^×*S*×*L*×*C*^ (3)

where *H* is the length of the forecast horizon and ***Q****_t_*_+*h*_ denotes the observed traffic state at the *h*-th forecast step, with *h* = 1, …, *H*. The forecasting task is represented by a parameterized mapping (*f_θ_*):

(4) $$\hat{Y_{t}}=f_{\theta }(X_{t}),\;\;f_{\theta }:R^{T\times S\times L\times C}\to R^{H\times S\times L\times C}$$

where *θ* denotes the learnable model parameters. The mapping jointly produces one flow estimate for every section–lane–class combination at each forecast step, so $\hat{Y_{t}}$ has the same structure as ***Y****_t_*.

This formulation assumes fixed monitoring locations and a fixed lane configuration over the study period. HV and CAV flows are observed separately for each section–lane unit and synchronized at a common aggregation interval. Only historical class-specific flow observations are used as model inputs.

## 4. Methodology

### 4.1. Overall Architecture

**Figure 3:**
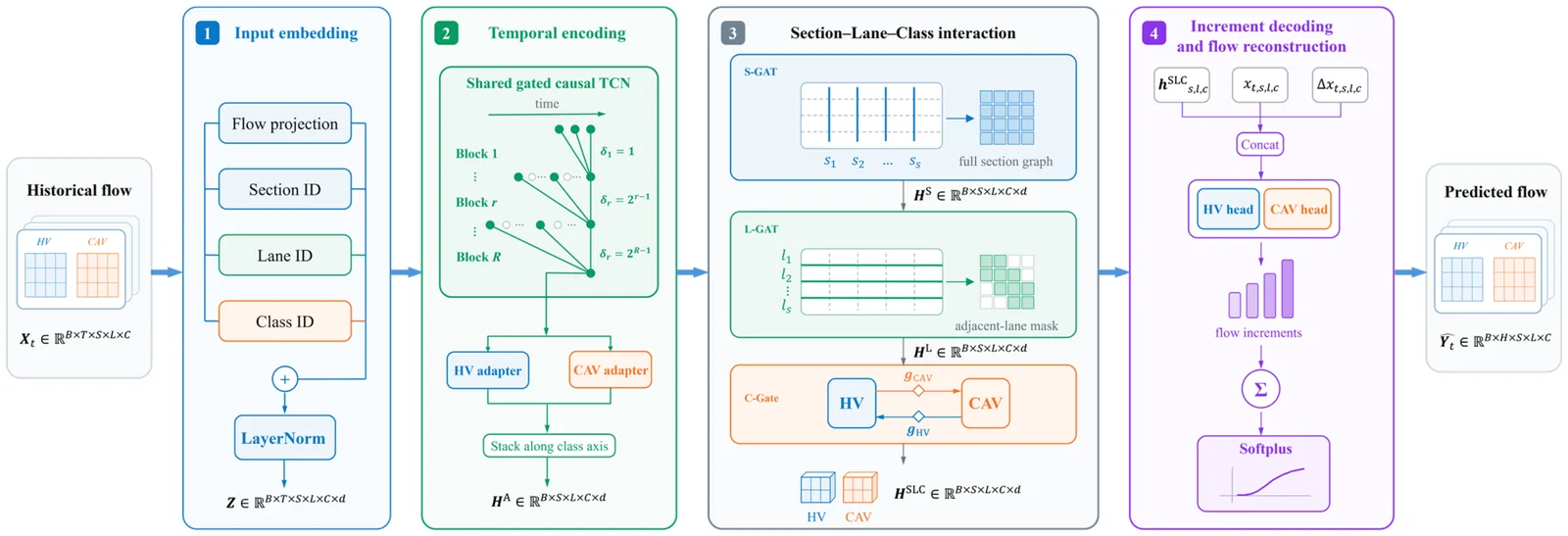
Overall architecture of the proposed TSLCN for lane-level mixed flow prediction. **Step 1: Input embedding.** Each scalar flow value is projected into a high-dimensional feature space and combined with the corresponding section, lane, and vehicle-class embeddings to encode the spatial location and vehicle class of each input series. **Step 2: Temporal encoding.** The embedded features are processed by a shared gated causal TCN. Dilated causal convolutions capture historical patterns over multiple temporal scales, while shared parameters learn temporal dynamics common to HV and CAV flows. Independently parameterized lightweight adapters then refine the representations for each vehicle class, yielding class-specific temporal features. **Step 3: Section–lane–class (SLC) interaction.** The interaction module applies fully connected section graph attention (S-GAT), adjacency-constrained lane graph attention (L-GAT), and a vehicle-class interaction gate (C-Gate) in sequence. For each fixed lane and vehicle class, S-GAT models relationships across monitoring sections. L-GAT captures lateral interactions between adjacent lanes within each section, whereas C-Gate exchanges information between the HV and CAV representations within each section–lane unit. The resulting features integrate temporal context with section, lane, and vehicle-class relationships. **Step 4: Increment decoding and flow reconstruction.** The final SLC representation is concatenated with the most recent standardized flow and its latest historical difference. Separate HV and CAV heads decode these features into standardized flow increments for all forecast horizons. The increments are accumulated from the latest standardized observation to reconstruct the future trajectories, which are then returned to the physical scale before Softplus produces positive flow forecasts.

Given the historical observation sequence defined in Section 3.1, TSLCN extracts temporal features while retaining the section, lane, and vehicle-class organization of the input. It then models longitudinal relationships across monitoring sections, lateral interactions between adjacent lanes, and local dependencies between HV and CAV flows to generate class-specific flow forecasts for every lane at each monitoring section. As shown in Figure 3, the architecture comprises four stages.

### 4.2. Input Embedding

Before embedding, each flow series is standardized using the mean and standard deviation computed from the training set, i.e., *x_t_*_,*s*,*l*,*c*_ = (*q_t_*_,*s*,*l*,*c*_ − *μ_s_*_,*l*,*c*_)/*σ_s_*_,*l*,*c*_, where *μ_s_*_,*l*,*c*_ and *σ_s_*_,*l*,*c*_ denote the corresponding training-set mean and standard deviation. A shared linear layer projects each standardized scalar flow *x_t_*_,*s*,*l*,*c*_ into a *d*-dimensional hidden space. Learnable section, lane, and vehicle-class embeddings are then added to encode the associated section, lane, and vehicle class, and the resulting representation is layer-normalized to obtain ***z****_t_*_,*s*,*l*,*c*_ ∈ ℝ*^d^* (Equation (5)).

(5) $$\begin{array}{c} z_{t,s,l,c}=\mathrm{LN}(W_{x}x_{t,s,l,c}+b_{x}+{e^{S}}_{s}+{e^{L}}_{l}+{e^{C}}_{c}) \end{array}$$

where *x_t_*_,*s*,*l*,*c*_ is the standardized flow of vehicle class *c* at time step *t*, monitoring section *s*, and lane *l*. The ***W***_x_ and ***b***_x_ symbols are the projection weight and bias. Embeddings ${e^{S}}_{s}$, ${e^{L}}_{l}$, and ${e^{C}}_{c}$ identify section *s*, lane *l*, and class *c*, respectively, and LN(·) denotes layer normalization.

The embedded observations form ***Z*** ∈ ℝ*^B^*^×^*^T^*^×^*^S^*^×^*^L^*^×^*^C^*^×^*^d^*, where *B* is the batch size and each time–section–lane–class entry has *d* features.

### 4.3. Temporal Encoding

**Figure 4:**
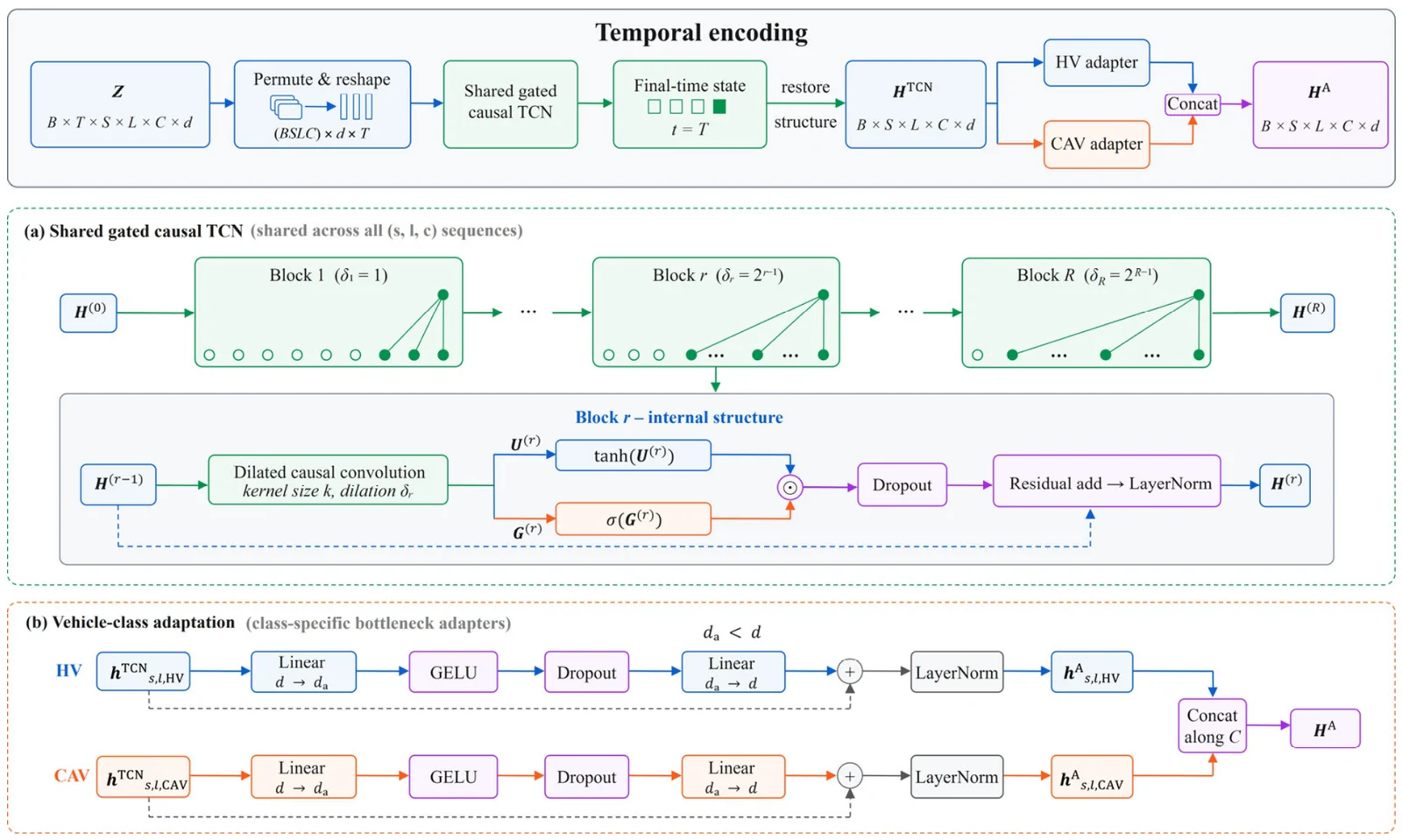
Architecture of the temporal encoding module.

Lane-level mixed traffic flows exhibit temporal continuity, while HV and CAV flows may follow different short-term dynamics. The temporal encoder therefore combines a shared gated causal TCN with class-specific adapters, as shown in Figure 4. The shared TCN uses common parameters across all section–lane–class series to learn general temporal patterns. Independently parameterized lightweight adapters then refine these shared representations to capture class-dependent temporal characteristics.

#### 4.3.1. Shared Gated Causal TCN

The shared TCN processes each section–lane–class series along time, with common parameters across all series. Causality excludes future observations, whereas dilation expands the receptive field efficiently [36]. Equation (6) summarizes the dilated convolution, tanh–sigmoid gate, dropout, residual connection, and layer normalization in block *r*.

(6) $$\begin{array}{c} {[U}^{\left(r\right)},G^{\left(r\right)}]={\mathrm{CausalConv}1D}_{k,\delta_{r}}(H^{\left(r-1\right)})\\ F^{\left(r\right)}=\tanh\left(U^{\left(r\right)}\right)⊙\sigma \left(G^{\left(r\right)}\right)\\ H^{\left(r\right)}=\mathrm{LN}(H^{\left(r-1\right)}+\mathrm{Dropout}(F^{\left(r\right)})) \end{array}$$

where *r* = 1, …, *R*, *R* and *k* are the number of blocks and kernel size and *δ_r_* is the dilation of block *r*. Variables ***U***^(*r*)^ and ***G***^(*r*)^ are the candidate and gate features. The initial sequence (***H***^(0)^) is obtained from ***Z*** by treating every section–lane–class series independently. Variables ***H***^(*r*−1)^ and ***H***^(*r*)^ are the block input and output, and ***F***^(*r*)^ is the gated residual feature produced by block *r*. Here, σ is the sigmoid function, ⊙ denotes elementwise multiplication, and CausalConv1D denotes a length-preserving one-dimensional causal convolution. The final historical state forms ***H***^TCN^ ∈ ℝ*^B^*^×*S*×*L*×*C*×*d*^; each vector at (*s*,*l*,*c*) summarizes one series over the input window. For block *r*, padding of *p_r_* = (*k* − 1)*δ_r_* time steps is applied, and the final *p_r_* outputs are removed so that the representation at time *t* depends only on observations available at or before *t*. For *R* blocks, the temporal receptive field is $R_{\mathrm{TCN}}=1+(k-1)\sum_{r=1}^{R}\delta_{r}$.

#### 4.3.2. Vehicle-Class Adaptation

To account for class-dependent temporal profiles and short-term variability, structurally identical but independently parameterized bottleneck residual adapters refine the shared HV and CAV representations [37]. For vehicle class *c*, the adaptation is defined in Equation (7).

(7) $${h^{A}}_{s,l,c}=\mathrm{LN}({h^{\mathrm{TCN}}}_{s,l,c}+{W^{A}}_{c,2}\mathrm{Dropout}(\mathrm{GELU}({W^{A}}_{c,1}{h^{\mathrm{TCN}}}_{s,l,c}+{b^{A}}_{c,1}))+{b^{A}}_{c,2})$$

where ${h^{\mathrm{TCN}}}_{s,l,c}$ and ${h^{A}}_{s,l,c}$ are the features before and after adaptation. Adapter parameters ${W^{A}}_{c,1}$, ${b^{A}}_{c,1}$, ${W^{A}}_{c,2}$, and ${b^{A}}_{c,2}$ are specific to vehicle class *c* and shared across all section–lane units within that class. The adapter maps *d* features to a bottleneck of dimension *d*_a_, applies Gaussian error linear unit (GELU) activation and dropout, and maps the residual correction back to *d* dimensions, with *d*_a_ < *d*. The adapted features form ***H***^A^ ∈ ℝ*^B^*^×*S*×*L*×*C*×*d*^ for SLC interaction.

### 4.4. Section–Lane–Class (SLC) Interaction

**Figure 5:**
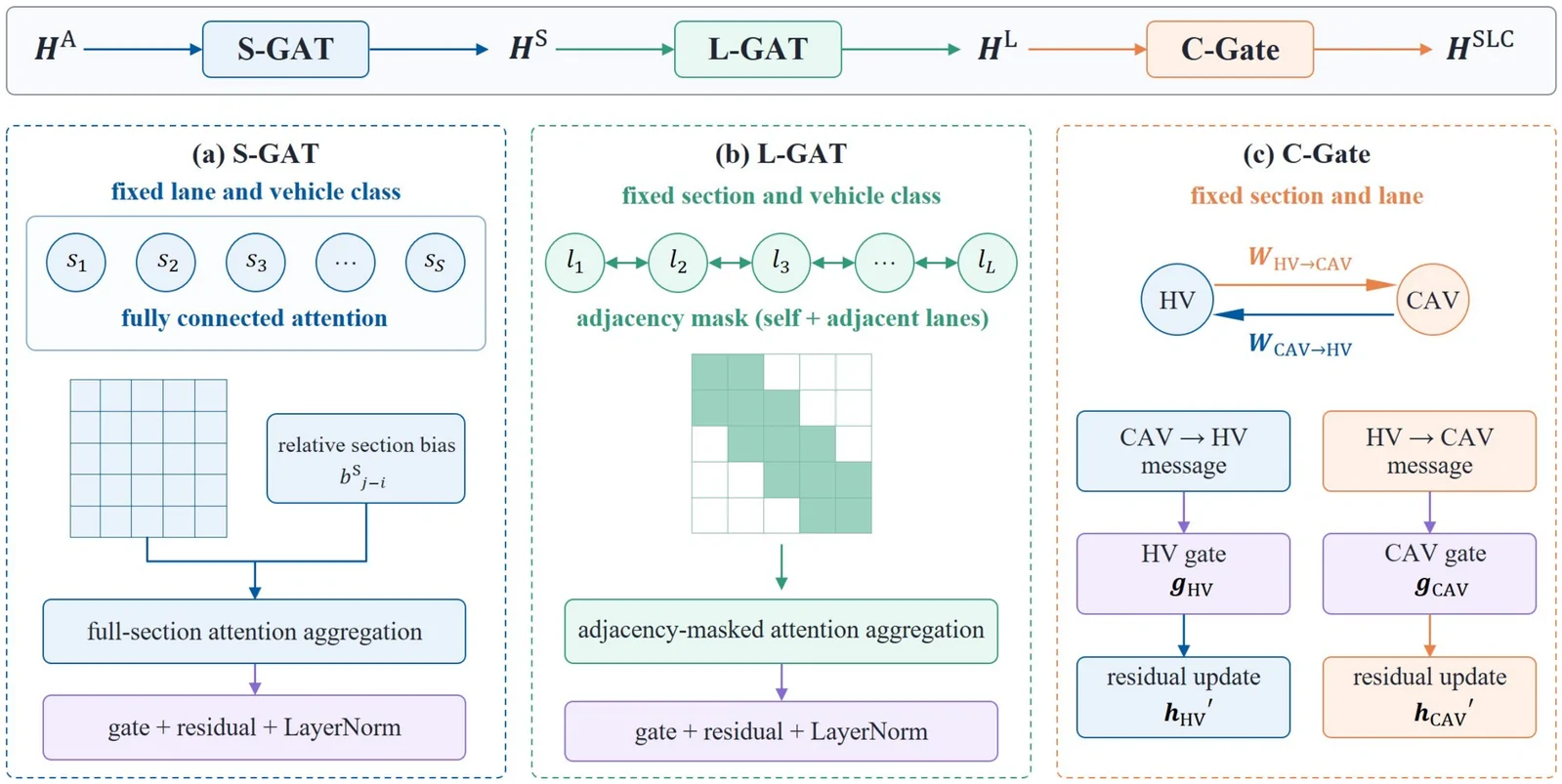
Architecture of SLC interaction module.

The SLC interaction module represents relationships across monitoring sections, between adjacent lanes, and between vehicle classes using S-GAT, L-GAT, and C-Gate, respectively (Figure 5).

Starting from the temporally encoded representation (***H***^A^), S-GAT produces the section-informed representation (***H***^S^). L-GAT then incorporates adjacent-lane information to obtain ***H***^L^, after which C-Gate models local cross-class dependence and yields ***H***^SLC^. The final representation is passed to the increment decoder. Indices held fixed within each component are omitted from Equations (8)–(10).

#### 4.4.1. Fully Connected Section Graph Attention (S-GAT)

For a fixed lane (*l*) and class (*c*), S-GAT represents the *S* monitoring sections as a fully connected directed graph with self-loops (Figure 5a). S-GAT uses scaled dot-product attention [38] within a graph-attention formulation [39], while a learned bias represents the signed positional offset between monitoring sections. Equation (8) defines the attention operation and gated residual update.

(8) $$\begin{array}{c} {q^{S}}_{i}={W^{S}}_{Q}{h^{A}}_{i},\;{k^{S}}_{j}={W^{S}}_{K}{h^{A}}_{j},\;{v^{S}}_{j}={W^{S}}_{V}{h^{A}}_{j}\;\\ {e^{S}}_{i,j}=\frac{{{q^{S}}_{i}}^{T}{k^{S}}_{j}}{\sqrt{d}}+{b^{S}}_{j-i},{\alpha^{S}}_{i,j}={\mathrm{Softmax}}_{j}\left({e^{S}}_{i,j}\right)\\ {m^{S}}_{i}={W^{S}}_{O}\sum_{j=1}^{S}{\alpha^{S}}_{i,j}{v^{S}}_{j}\\ {g^{S}}_{i}=\sigma \left({W^{S}}_{g}\left[{h^{A}}_{i}∥{m^{S}}_{i}\right]+{b^{S}}_{g}\right)\\ {h^{S}}_{i}=\mathrm{LN}({h^{A}}_{i}+\mathrm{Dropout}({g^{S}}_{i}⊙{m^{S}}_{i})) \end{array}$$

where *i* and *j* index target and source sections, respectively; ${q^{S}}_{i}$, ${k^{S}}_{j}$, and ${v^{S}}_{j}$ are their query, key, and value vectors; and ${W^{S}}_{Q}$, ${W^{S}}_{K}$, and ${W^{S}}_{V}$ are the associated projections. Variable ${e^{S}}_{i,j}$ is the unnormalized score, *d* is the query/key dimension, and ${b^{S}}_{j-i}$ is a learned bias for signed displacement *j* − *i*. Variable ${\alpha^{S}}_{i,j}$ is the normalized weight, ${m^{S}}_{i}$ is the aggregated message, and ${g^{S}}_{i}$ is the message gate. Matrices ${W^{S}}_{O}$ and ${W^{S}}_{g}$, together with the gate bias, are learnable. Dropout, a residual update, and layer normalization produce the section outputs (***H***^S^ ∈ ℝ*^B^*^×*S*×*L*×*C*×*d*^).

#### 4.4.2. Adjacency-Constrained Lane Graph Attention (L-GAT)

For a fixed section (*s*) and class *c*, L-GAT represents lanes as graph nodes and permits interaction only with the target lane and its immediate neighbors (Figure 5b). The resulting banded mask excludes nonexistent boundary neighbors and nonadjacent lanes.

L-GAT uses projections independent of S-GAT. Attention is normalized only over the permitted neighborhood, assigning zero weight to nonadjacent lanes, as defined in Equation (9).

(9) $$\begin{array}{c} {q^{L}}_{i}={W^{L}}_{Q}{h^{S}}_{i},\;{k^{L}}_{j}={W^{L}}_{K}{h^{S}}_{j},\;{v^{L}}_{j}={W^{L}}_{V}{h^{S}}_{j}\\ {e^{L}}_{i,j}=\frac{{{q^{L}}_{i}}^{T}{k^{L}}_{j}}{\sqrt{d}},j\in {N^{L}}_{i}\\ {\alpha^{L}}_{i,j}=\frac{\exp({e^{L}}_{i,j})}{\sum_{j^{\prime }\in {N^{L}}_{i}}\exp({e^{L}}_{i,j^{\prime }})},j\in {N^{L}}_{i}\\ {m^{L}}_{i}={W^{L}}_{O}\sum_{j\in {N^{L}}_{i}}{\alpha^{L}}_{i,j}{v^{L}}_{j}\\ g_{i}^{L}=\sigma (W_{g}^{L}[h_{i}^{S}∥m_{i}^{L}]+b_{g}^{L})\\ h_{i}^{L}=\mathrm{LN}(h_{i}^{S}+\mathrm{Dropout}(g_{i}^{L}⊙m_{i}^{L})) \end{array}$$

where $N_{i}^{L}$ contains lane *i* and its existing immediate neighbors. Vectors $q_{i}^{L}$, $k_{j}^{L}$, and $v_{j}^{L}$ are the query, key, and value representations, respectively. Variables $e_{\mathrm{ij}}^{L}$ and $\alpha_{\mathrm{ij}}^{L}$ denote the attention score and its normalized weight, while $m_{i}^{L}$ is the aggregated lane message. The gate ($g_{i}^{L}$) regulates the contribution of this message before the residual update and layer normalization produce $h_{i}^{L}$. Although the adjacency relation is symmetric, $\alpha_{\mathrm{ij}}^{L}$ and $\alpha_{\mathrm{ji}}^{L}$ may differ because they are conditioned on different target queries. The lane-informed representations form ***H****^L^* ∈ ℝ*^B^*^×*S*×*L*×*C*×*d*^.

#### 4.4.3. Vehicle-Class Interaction Gate (C-Gate)

Within each section–lane unit, C-Gate exchanges transformed messages between the HV and CAV representations, with gates controlling the contribution of the received information (Figure 5c). The update is defined in Equation (10).

(10) $$\begin{array}{c} m_{\mathrm{CAV}\to \mathrm{HV}}=W_{\mathrm{CAV}\to \mathrm{HV}}h_{\mathrm{CAV}},m_{\mathrm{HV}\to \mathrm{CAV}}=W_{\mathrm{HV}\to \mathrm{CAV}}h_{\mathrm{HV}}\\ g_{\mathrm{HV}}=\sigma ({W^{\mathrm{HV}}}_{g}[h_{\mathrm{HV}}∥m_{\mathrm{CAV}\to \mathrm{HV}}]+{b^{\mathrm{HV}}}_{g})\\ g_{\mathrm{CAV}}=\sigma ({W^{\mathrm{CAV}}}_{g}[h_{\mathrm{CAV}}∥m_{\mathrm{HV}\to \mathrm{CAV}}]+{b^{\mathrm{CAV}}}_{g})\\ {h_{\mathrm{HV}}}^{\prime }=\mathrm{LN}(h_{\mathrm{HV}}+\mathrm{Dropout}(g_{\mathrm{HV}}⊙m_{\mathrm{CAV}\to \mathrm{HV}}))\\ {h_{\mathrm{CAV}}}^{\prime }=\mathrm{LN}(h_{\mathrm{CAV}}+\mathrm{Dropout}(g_{\mathrm{CAV}}⊙m_{\mathrm{HV}\to \mathrm{CAV}})) \end{array}$$

where variables ***h***_HV_ and ***h***_CAV_ are the HV and CAV features produced by L-GAT. Messages ***m***_CAV→HV_ and ***m***_HV→CAV_ are generated by the corresponding linear maps (***W***_CAV→HV_ and ***W***_HV→CAV_). Gates ***g***_HV_ and ***g***_CAV_ control the received information. The associated projection matrices (${W^{\mathrm{HV}}}_{g}$ and ${W^{\mathrm{CAV}}}_{g}$), together with their biases, are learnable. Variables ${h_{\mathrm{HV}}}^{\prime }$ and ${h_{\mathrm{CAV}}}^{\prime }$ are the updated class-specific features.

The two outputs are combined into the final SLC representation (***H***^SLC^ ∈ ℝ*^B^*^×*S*×*L*×*C*×*d*^).

### 4.5. Increment Decoding and Flow Reconstruction

**Figure 6:**
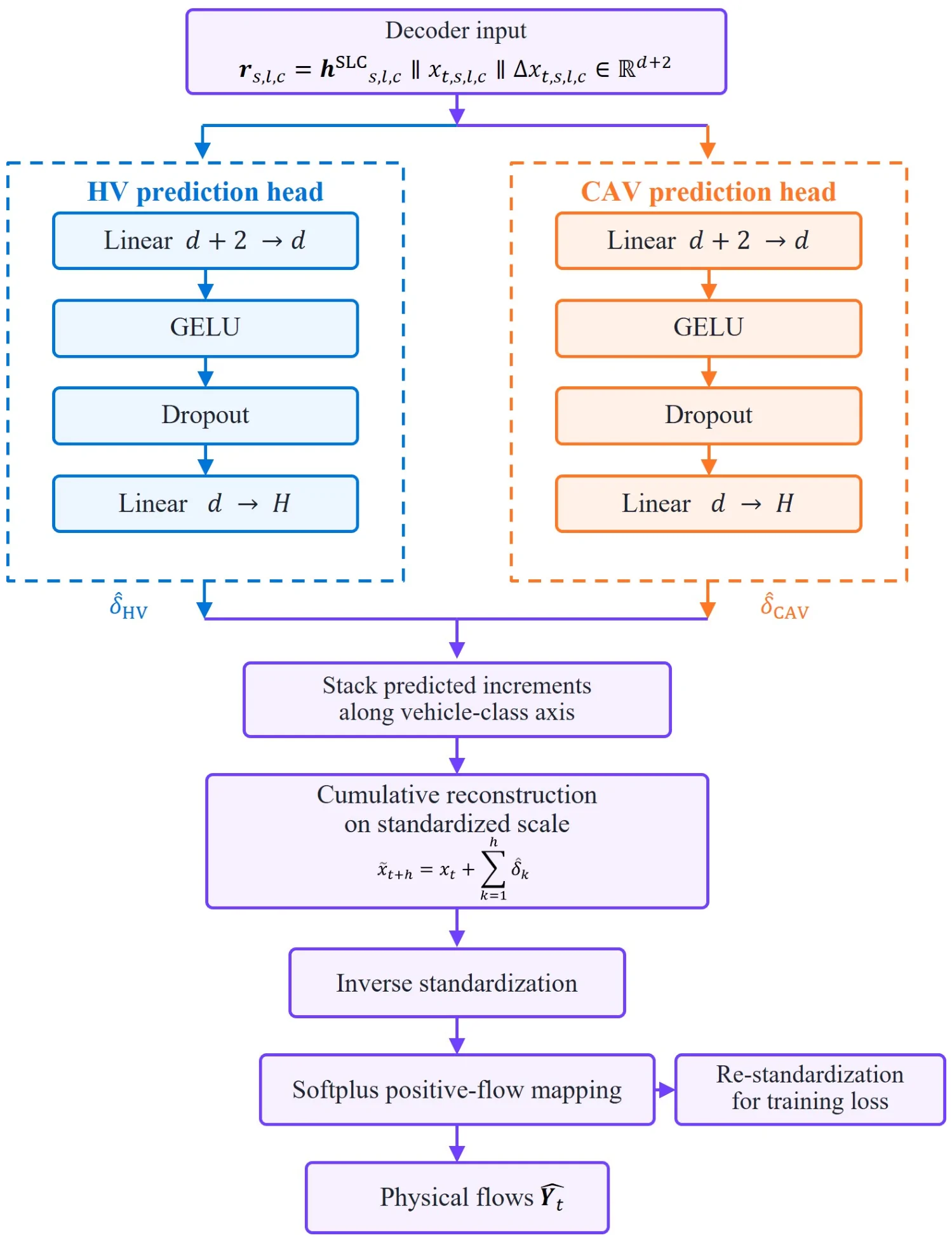
Process of increment decoding and flow reconstruction.

For each section–lane–class unit, the decoder concatenates ${h^{\mathrm{SLC}}}_{s,l,c}$ with the last standardized flow and latest historical difference. Separate heads then predict standardized increments for all *H* horizons. The increment decoding and flow reconstruction process is illustrated in Figure 6.

For monitoring of section *s*, lane *l*, and vehicle class *c*, the recent flow change, decoder feature, predicted increments, and cumulative reconstruction are defined in Equation (11).

(11) $$\begin{array}{c} {\Delta x}_{t,s,l,c}=x_{t,s,l,c}-x_{t-1,s,l,c}\\ r_{s,l,c}={h^{\mathrm{SLC}}}_{s,l,c}∥x_{t,s,l,c}∥{\Delta x}_{t,s,l,c}\\ {\hat{\delta }}_{s,l,c}={\mathrm{Head}}_{c}\left(r_{s,l,c}\right)=[{\hat{\delta }}_{1,s,l,c},\ldots ,{\hat{\delta }}_{H,s,l,c}]\in R^{H}\\ {\tilde{x}}_{t+h,s,l,c}=x_{t,s,l,c}+\sum_{k=1}^{h}{\hat{\delta }}_{k,s,l,c},\;h=1,\ldots ,H \end{array}$$

where Δ*x_t_*_,*s*,*l*,*c*_ is the latest historical difference and ***r****_s_*_,*l*,*c*_ is the decoder feature. The notation ${\mathrm{Head}}_{c}(\cdot ),{\hat{\delta }}_{k,s,l,c}$ denotes the class-specific head and its *k*-th increment, respectively, and ${\tilde{x}}_{t+h,s,l,c}$ is the candidate standardized flow at horizon *h*. The HV and CAV heads use the same two-layer feed-forward structure with GELU activation and dropout, with separate parameters for the two classes. Each head produces all *H* forecast increments simultaneously in a single forward pass, following a direct multi-output strategy.

The predicted future increments may be positive or negative, allowing the reconstructed flow to increase or decrease. The accumulated candidates are first returned to the physical scale using statistics computed from the training set. Softplus then maps them to strictly positive physical flows. The resulting flows are restandardized before the training loss is evaluated, as shown in Equation (12).

(12) $$\begin{array}{c} {\tilde{q}}_{t+h,s,l,c}=\mu_{s,l,c}+\sigma_{s,l,c}{\tilde{x}}_{t+h,s,l,c}\\ {\hat{q}}_{t+h,s,l,c}=\mathrm{Softplus}({\tilde{q}}_{t+h,s,l,c})>0\\ {\hat{x}}_{t+h,s,l,c}=\frac{{\hat{q}}_{t+h,s,l,c}-\mu_{s,l,c}}{\sigma_{s,l,c}} \end{array}$$

where *μ_s_*_,*l*,*c*_ and *σ_s_*_,*l*,*c*_ are the training-set mean and standard deviation for the corresponding flow series. Variables ${\tilde{q}}_{t+h,s,l,c}$, ${\hat{q}}_{t+h,s,l,c}$, and ${\hat{x}}_{t+h,s,l,c}$ denote the candidate physical-scale flow, the strictly positive physical prediction, and the restandardized prediction, respectively.

### 4.6. Joint Training Objective

The objective combines a flow-level loss with a change loss, both based on the Smooth L1 form of the Huber loss [40,41]. Equation (13) defines the flow-level loss for vehicle class *c*.

(13) $$\begin{array}{c} \rho_{\beta }(e)=\left\{\begin{array}{c} \frac{e^{2}}{2\beta },\;\left|e\right|<\beta \\ \left|e\right|-\frac{\beta }{2},\;\mathrm{otherwise} \end{array}\right.\\ {L^{c}}_{\mathrm{lev}}=\frac{1}{\mathrm{BHSL}}\sum_{b,h,s,l}\rho_{\beta }\left({\hat{x}}_{b,h,s,l,c}-x_{b,h,s,l,c}\right)\\ L_{\mathrm{lev}}=\frac{1}{C}\sum_{c=1}^{C}{L^{c}}_{\mathrm{lev}} \end{array}$$

where *e* is the scalar prediction error and $\hat{x}$ and *x* are the standardized prediction and target. Losses ${L^{c}}_{\mathrm{lev}}$ and ℒ_lev_ are the class-specific and class-averaged flow-level losses; the latter weights the two classes equally, regardless of prevalence. The threshold of *β* > 0 separates the quadratic and linear regions of the Huber loss.

The change loss compares first-order differences at successive forecast steps (Equation (14)); for step 0, both sequences begin from the final historical observation.

(14) $$\begin{array}{c} {L^{c}}_{\Delta }=\frac{1}{\mathrm{BHSL}}\sum_{b,h,s,l}\rho_{\beta }(({\hat{x}}_{b,h,s,l,c}-{\hat{x}}_{b,h-1,s,l,c})-(x_{b,h,s,l,c}-x_{b,h-1,s,l,c}))\\ L_{\Delta }=\frac{1}{C}\sum_{c=1}^{C}{L^{c}}_{\Delta } \end{array}$$

The final objective combines the two losses (Equation (15)).

(15) $$L=L_{\mathrm{lev}}+\lambda_{\Delta }L_{\Delta }$$

where *λ*_Δ_ ≥ 0 controls the contribution of the change loss. The shared TCN, class-specific adapters, SLC interaction module, and decoder are optimized jointly using this objective.

## 5. Experiments

### 5.1. Experimental Setup

#### 5.1.1. Data Sources

The evaluation dataset was generated in Simulation of Urban MObility (SUMO) from field-observed traffic demand, enabling HV and CAV flows to be recorded separately at lane level. SUMO permits vehicle-dynamics and behavioral parameters to be specified for automated-vehicle and freeway simulation studies [42,43].

**Figure 7:**
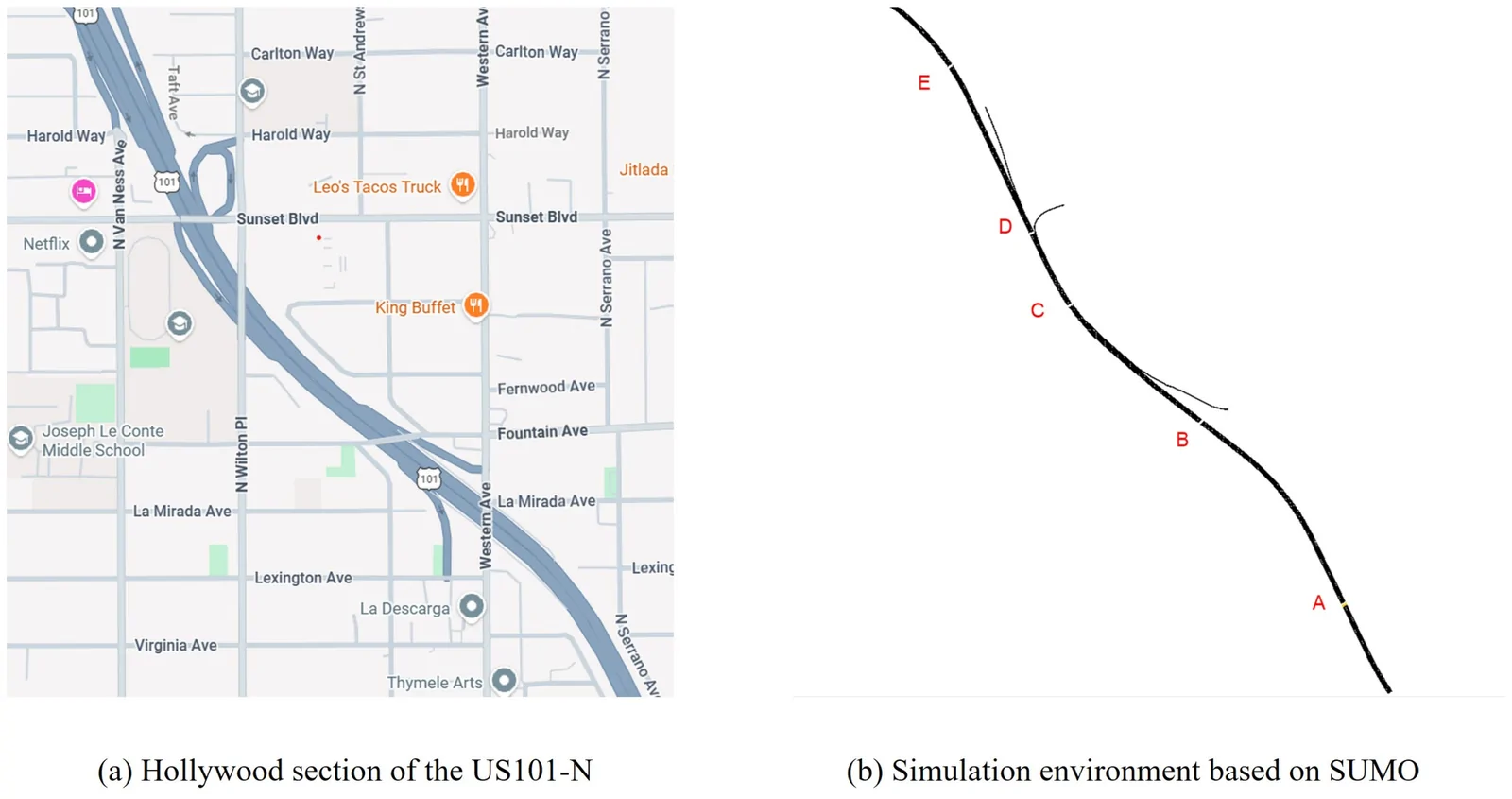
Study corridor and SUMO simulation environment.

A 2.2-km northbound segment of US 101 in Hollywood, Los Angeles, was selected as the study corridor (Figure 7). The segment comprises four mainline lanes, with a speed limit of 70 mph, and includes one entrance ramp and two exit ramps. It experiences recurrent peak-period congestion and pronounced lane-flow variability. Because passenger cars dominate the observed traffic and heavy vehicles are rare, only passenger cars, represented as either HVs or CAVs, were modeled.

Traffic volumes recorded by the Caltrans Performance Measurement System (PeMS) from 1 to 31 May 2018 were used to define the baseline demand. Five monitoring sections, labeled A–E from south to north, were placed along the corridor, with each section covering four lanes. HV and CAV flows were recorded separately at 5-min intervals. The dataset contains 8928 consecutive time steps and 40 flow variables at each step, corresponding to five sections, four lanes, and two vehicle classes.

#### 5.1.2. Simulation Configuration and Scenario Design

The simulated traffic comprised HVs and CAVs. Simulations were conducted in SUMO version 1.22.0 with a simulation step of 0.1 s. Vehicle parameters were specified with reference to previous mixed-traffic simulation studies [7,44], as summarized in Table 2. HV and CAV longitudinal behavior was represented using the Intelligent Driver Model (IDM) and Cooperative Adaptive Cruise Control (CACC), respectively. Both classes used the LC2013 lane-changing model with class-specific parameters.

**Figure 8:**
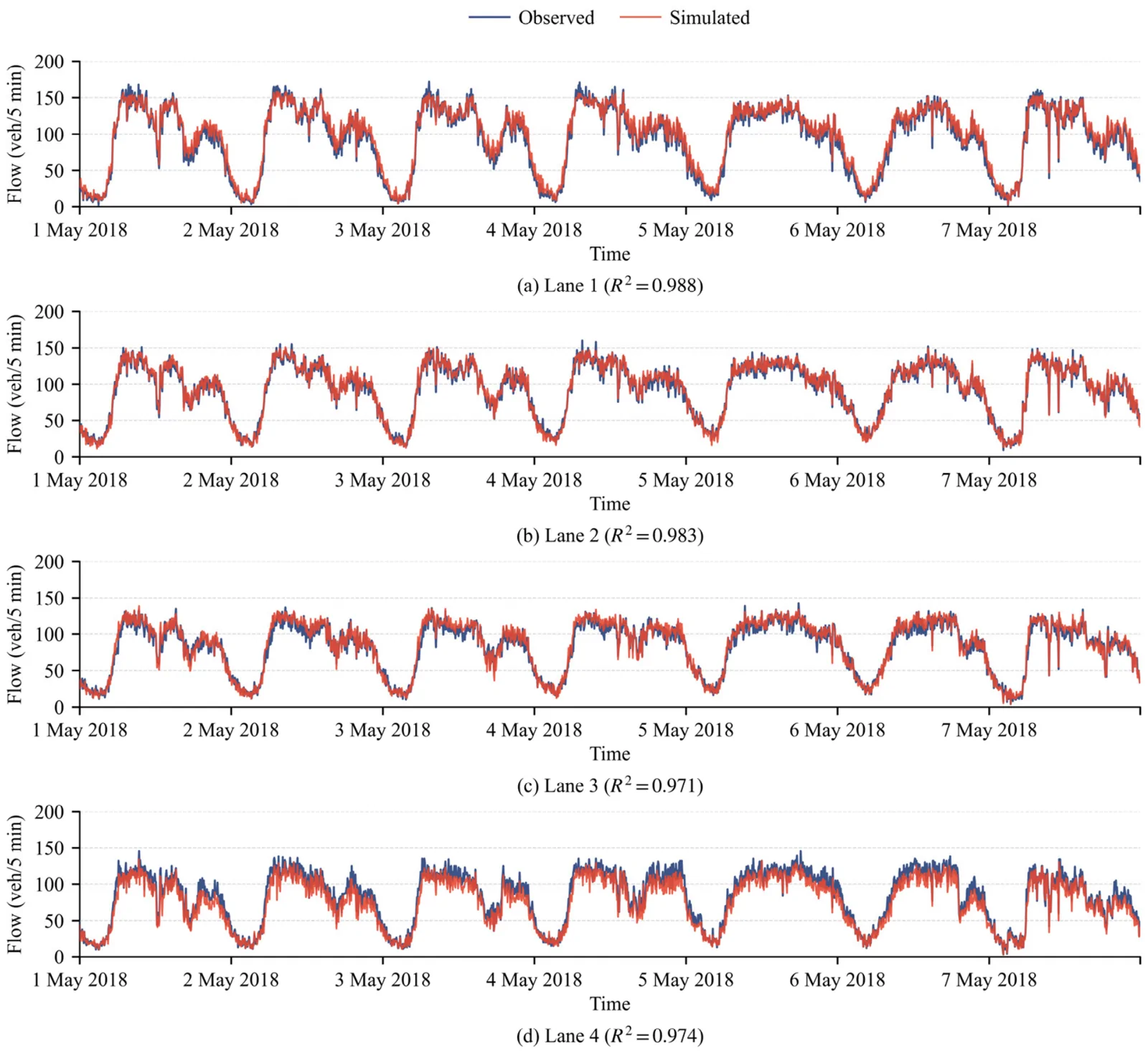
Observed and simulated 5-min lane flows at internal monitoring section C under the all-HV base scenario from 1 to 7 May 2018.

The observed 5-min PeMS counts were used to specify time-varying demands at the upstream mainline boundary and entrance ramp. Vehicle arrivals at each entry were modeled as a Poisson process with a piecewise-constant rate over successive 5-min intervals. For entry *e* and interval *t*, the arrival rate was defined as *λ_e_*_,*t*_ = *q_e_*_,*t*_/300 vehicles per second, where *q_e_*_,*t*_ is the corresponding observed vehicle count. Route shares for the two exit ramps were determined from their observed 5-min flows, with the remaining vehicles assigned to the through route. These demand and route settings were first applied to the all-HV base scenario. After a 10-min warm-up, simulated lane flows at the internal monitoring section (C) were aggregated at the same 5-min resolution and compared with the corresponding PeMS observations from 1 to 7 May 2018 (Figure 8). Lanes were numbered from the innermost lane (Lane 1) to the outermost lane (Lane 4).

Across the four lanes, the coefficients of determination calculated from the paired observed and simulated 5-min flows ranged from 0.971 to 0.988. Seven traffic-composition scenarios were generated with CAV penetration rates of 0%, 10%, 30%, 50%, 70%, 90%, and 100%. The 0% and 100% scenarios represent pure HV and pure CAV traffic, respectively, while vehicles in the intermediate scenarios were assigned as CAVs according to the prescribed penetration rate. Across these penetration scenarios, total demand, route assignment, and the remaining simulation settings were held constant, and the same random seeds were used to control simulation variability. The 30% scenario was used for the detailed baseline comparison and ablation analysis, while all seven scenarios were used to examine forecasting performance across different traffic compositions. The sensitivity analysis was also conducted under the 30% scenario by varying selected car-following and lane-changing parameters individually around their reference settings in Table 2, with demand, routing, and all other simulation parameters held fixed.

#### 5.1.3. Baseline Models

Seven baseline models were selected to cover temporal, network-wide spatiotemporal, and lane-level forecasting formulations. ARIMA [45] and GRU [46] provide statistical and neural temporal references without explicit spatial interaction. GATCN [20,39] and Graph WaveNet [21] represent graph-based forecasting with prescribed and learned connectivity, respectively. The Spatio-Temporal Adaptive Embedding Transformer (STAEformer) [47] provides a global self-attention-based comparison using adaptive spatiotemporal embeddings. Two methods developed specifically for lane-level forecasting were also included. The multi-lane convolutional long short-term memory network (Multi-Lane ConvLSTM) [48] models local continuity over the section–lane grid, whereas STA-ED [35] uses target-specific attention to forecast heterogeneous lane-level flows.

All baselines used flow as the input and forecasted the same 5 × 4 × 2 section–lane–class targets. ARIMA and GRU processed the 40 scalar flow series independently, although the GRU shared its parameters across series. GATCN, Graph WaveNet, and STAEformer represented the 20 section–lane units as spatial nodes, with HV and CAV flows used as node features. Multi-Lane ConvLSTM arranged the observations as a 5 × 4 grid with two vehicle-class channels. STA-ED used a separate encoder–decoder for each of the 40 prediction targets while attending to all input series. Dropout was set to 0.1 in GRU, GATCN, Graph WaveNet, STAEformer, and Multi-Lane ConvLSTM. Table 3 summarizes the model-specific configurations.

#### 5.1.4. Evaluation Metrics

Prediction accuracy was evaluated using mean absolute error (MAE), root mean square error (RMSE), and weighted absolute percentage error (WAPE):

(16) $$\mathrm{MAE}=\frac{1}{n}\sum_{i=1}^{n}\left|y_{i}-{\hat{y}}_{i}\right|$$

(17) $$\mathrm{RMSE}=\sqrt{\frac{1}{n}\sum_{i=1}^{n}{\left(y_{i}-{\hat{y}}_{i}\right)}^{2}}$$

(18) $$\mathrm{WAPE}=\frac{\sum_{i=1}^{n}\left|y_{i}-{\hat{y}}_{i}\right|}{\sum_{i=1}^{n}\left|y_{i}\right|}\times 100\%$$

MAE summarizes the average absolute error, whereas RMSE assigns greater weight to large deviations. WAPE normalizes the total absolute error by the total observed flow. Its global denominator remains well defined when individual lane–class observations are zero and provides a scale-normalized complement to MAE and RMSE. Lower values indicate better predictive performance for all three metrics.

Horizon-specific differences in MAE were examined using two-sided Diebold–Mariano tests [49]. At each forecast horizon, the baseline with the lowest mean MAE was used as the comparator. For each prediction origin, the absolute errors were averaged across the independent runs, monitoring sections, lanes, and vehicle classes, yielding one loss series for each model and forecast horizon. The loss differential was calculated as the baseline loss minus the TSLCN loss so that a positive mean difference indicated a lower MAE for TSLCN. Serial dependence in the loss differentials was accounted for using Newey–West heteroskedasticity- and autocorrelation-consistent standard errors with Bartlett weights [50] and lag *h* − 1, where *h* denotes the forecast step. The Harvey–Leybourne–Newbold small-sample correction was applied to the test statistic [51], and the corresponding HAC standard errors were used to calculate 95% confidence intervals for the mean MAE differences. The resulting *p*-values were adjusted across the forecast horizons using the Holm procedure [52], with statistical significance assessed at *α* = 0.05.

#### 5.1.5. Data Partitioning, Training, and Implementation

The dataset was divided chronologically into training, validation, and test sets using a 70%/15%/15% ratio. The mean and standard deviation of each flow series were computed from the training set only, then applied unchanged to the validation and test sets. After pre-experiment optimization, each sample used the preceding 15 observations, equivalent to 75 min, to predict the next three 5-min intervals. Windows were assigned to partitions according to their forecast target times. After window construction, the partitions contained 6232 training, 1337 validation, and 1338 test samples. Performance was evaluated separately at horizons of 5, 10, and 15 min.

The TSLCN implementation used a hidden dimension of 32 and a dropout rate of 0.1. Its temporal encoder contained three gated causal convolution blocks with a kernel size of 3 and dilation factors of 1, 2, and 4, yielding a receptive field of 15 observations. Each class-specific adapter used an eight-unit bottleneck. S-GAT and L-GAT each contained a single attention head with 32-dimensional query, key, and value projections. The cumulative-increment decoder generated all three forecast horizons in a single forward pass. The remaining optimization settings were shared across the neural models, as summarized in Table 4.

All experiments were implemented in Python 3.10 with PyTorch 2.6.0 and executed using 12 CPU threads on an Intel Core i5-13600KF processor.

### 5.2. Comparison with Baseline Models

#### 5.2.1. Overall Performance and Statistical Comparison

Table 5 reports the overall forecasting performance obtained by pooling predictions over all test samples, forecast horizons, monitoring sections, lanes, and vehicle classes. Results for the neural models are presented as the mean ± SD over five random seeds. TSLCN achieved the lowest mean error for all three metrics, with an MAE of 5.67 ± 0.006, an RMSE of 7.74 ± 0.009, and a WAPE of 11.62 ± 0.012%. Across the seven baselines, MAE ranged from 5.75 to 6.71, RMSE from 7.83 to 8.93, and WAPE from 11.79% to 13.75%. Multi-Lane ConvLSTM recorded the lowest baseline MAE and WAPE, whereas Graph WaveNet recorded the lowest baseline RMSE. Calculated from the unrounded means, TSLCN reduced MAE by 1.45% relative to Multi-Lane ConvLSTM, RMSE by 1.18% relative to Graph WaveNet, and WAPE by 1.45% relative to Multi-Lane ConvLSTM.

ARIMA and GRU primarily relied on the temporal evolution of the flow series and produced the two largest aggregate errors, indicating that temporal information alone did not fully represent traffic propagation and lane-level flow redistribution. The spatial baselines incorporated traffic conditions beyond the target series and generally achieved lower errors. GATCN used topology-constrained graph attention to aggregate information from physically connected locations, allowing upstream, downstream, and neighboring-lane states to contribute according to their relevance to the current prediction. STAEformer applied global spatial self-attention across all section–lane nodes, enabling nonlocal traffic correlations to be represented, and recorded the lowest baseline MAE at 5 min. STA-ED assigned target-specific weights to multiple input series, accounting for the varying relevance of lane and vehicle-class observations to each prediction target. Graph WaveNet learned directed adaptive connections that reflected the asymmetric propagation of traffic states along the freeway and obtained the lowest baseline RMSE. Multi-Lane ConvLSTM applied convolutional recurrence to the section–lane grid, preserving local continuity across neighboring sections and lanes and achieving the best aggregate performance among the baselines. TSLCN represented the main traffic relationships according to their respective interaction ranges. Section attention captured longitudinal propagation along the corridor, lane attention represented flow redistribution between adjacent lanes, and C-Gate modeled local coupling between HV and CAV flows. The shared temporal encoder captured patterns common to both vehicle classes, while the class-specific adapters retained differences in their short-term evolution. This alignment between traffic processes and model operations helps explain the lowest overall MAE, RMSE, and WAPE obtained by TSLCN.

Table 6 compares TSLCN with the baseline yielding the lowest mean MAE at each forecast horizon. The strongest baseline changed from STAEformer at 5 min to Graph WaveNet at 10 min and Multi-Lane ConvLSTM at 15 min. Across the three horizons, TSLCN reduced MAE by 0.062–0.093 vehicles per 5 min, corresponding to relative reductions of 1.04–1.70%, with the largest reduction observed at 5 min. All 95% HAC confidence intervals excluded zero. The two-sided Diebold–Mariano tests remained significant after Holm adjustment, with *p* < 0.001 at 5 and 10 min and *p* = 0.006 at 15 min. The reductions were therefore modest in magnitude but statistically significant across all three forecast horizons.

#### 5.2.2. Performance Across Vehicle Classes, Lanes, and Forecast Horizons

**Figure 9:**
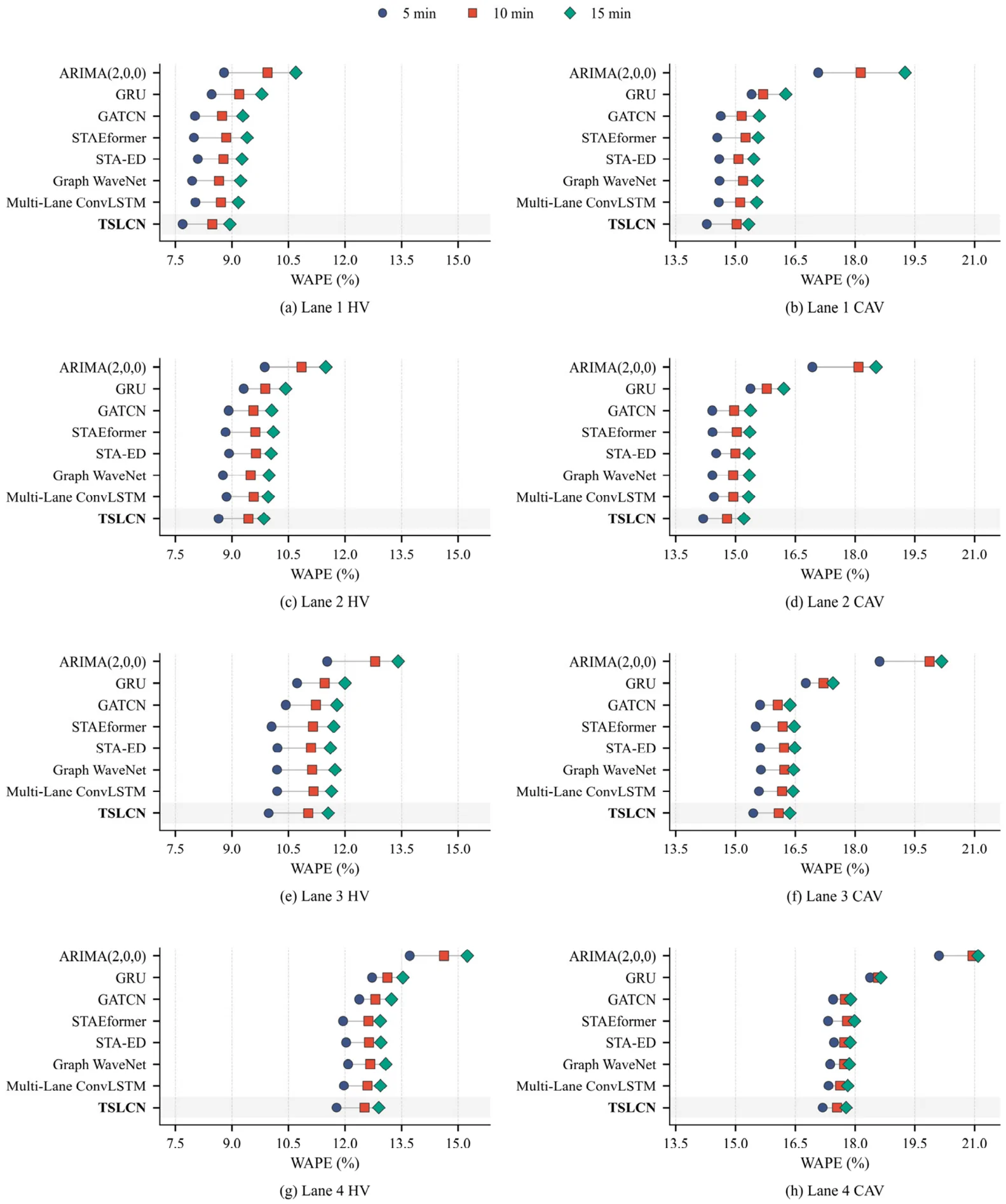
Lane-specific multi-horizon WAPE of the compared models for HVs and CAVs at 30% CAV penetration.

Table 7 reports the lane-specific multi-step forecasting results for HVs and CAVs, while Figure 9 summarizes WAPE by lane, vehicle class, and forecast horizon. The identity of the strongest competing baseline varied with lane, vehicle class, forecast horizon, and evaluation metric. Across the same 72 lane–class–horizon–metric combinations, TSLCN recorded the lowest five-run mean in 68 cases (94.4%). These leading results covered both vehicle classes, all four lanes, and all three forecast horizons. The four exceptions all concerned the Lane 3 CAV forecasts at 10 or 15 min. At 10 min, GATCN obtained an MAE of 4.58, an RMSE of 6.02, and a WAPE of 16.06%, compared with 4.59, 6.03, and 16.08% for TSLCN. The remaining exception was the 15-min RMSE, which was 6.13 for GATCN and 6.14 for TSLCN. The slight advantage of GATCN may be related to the position of Lane 3, which adjoins Lane 4, the ramp-connected outer lane. Its CAV flow may therefore be influenced by localized redistribution associated with ramp-related merging, diverging, and lane-changing movements.

Prediction errors generally increased from 5 to 15 min for both vehicle classes across all four lanes. As the forecast horizon lengthened, the traffic state at the target time was less strongly constrained by recent observations, while future variations in traffic demand, lane-changing activity, and queue propagation became increasingly difficult to infer from the fixed historical window. TSLCN recorded the lowest mean error in all 24 lane–class–metric comparisons at 5 min and in 44 of the 48 comparisons at 10 and 15 min.

The two vehicle classes showed different absolute and relative error patterns. Averaging the 12 lane–horizon results for TSLCN gave an MAE of 6.78 for HVs and 4.56 for CAVs, with corresponding WAPEs of 10.23% and 15.77%. At 30% CAV penetration, the lower CAV flow volumes were associated with smaller absolute errors. As WAPE is normalized by the total observed flow, the smaller denominator resulted in higher relative errors for CAVs. With fewer CAVs observed in each lane and time interval, changes in the arrival timing or lane allocation of only a few vehicles can account for a larger proportion of the recorded flow, producing greater relative fluctuations. The lower CAV MAE therefore reflects, at least in part, the smaller scale of the predicted series.

Lane-specific differences were most evident in WAPE, particularly between the innermost and outermost lanes. For HVs, TSLCN WAPE ranged from 7.69% to 8.94% in Lane 1 and from 11.77% to 12.90% in Lane 4. For CAVs, the corresponding ranges were 14.28–15.33% and 17.19–17.78%. Lane 1 mainly carried through traffic, whereas Lane 4 was directly connected to the ramps and affected by merging, diverging, and mandatory lane changes. The combination of lower flow volumes and greater short-term fluctuations was associated with higher relative errors in the outermost lane. CAV WAPE was slightly lower in Lane 2 than in Lane 1 and then increased toward Lane 4, indicating that the lane pattern was not strictly monotonic.

**Figure 10:**
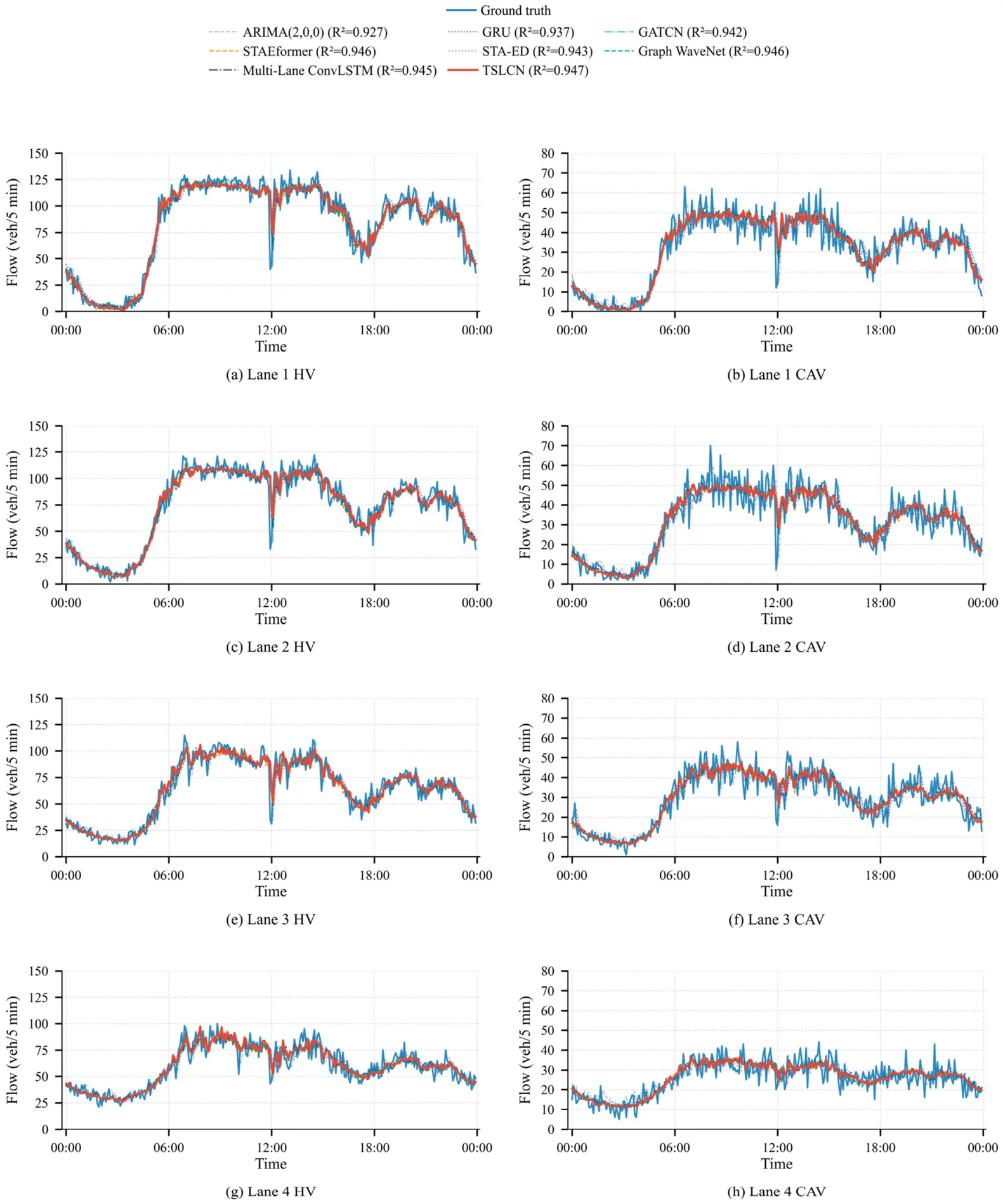
Ground-truth and 5-min-ahead predicted HV and CAV flows across four lanes at Section C under 30% CAV penetration on 29 May 2018.

Located within the weaving area, Section C experiences frequent merging, diverging, and lane-changing movements, producing pronounced short-term variations across lanes and vehicle classes. Figure 10 compares the 5-min-ahead predictions with the simulated reference flows at this section on 29 May 2018. All models reproduced the broad daily flow pattern, while visible deviations were concentrated around rapid changes and short-lived fluctuations. TSLCN followed the sustained peaks, troughs, and turning points of both HV and CAV flows, but it smoothed some high-frequency variations and did not fully reproduce several abrupt flow drops around midday. These discrepancies were more apparent in the CAV series, which exhibited greater short-term fluctuations. No consistent deterioration from the inner to the outer lane was visually evident in this example, suggesting that the principal deviations were associated more with abrupt temporal changes than with lane position. For each model, the summary R^2^ was calculated by pooling the observations across the eight lane–class panels. TSLCN achieved the highest pooled R^2^ of 0.947, compared with 0.946 for STAEformer and Graph WaveNet, 0.945 for Multi-Lane ConvLSTM, 0.943 for STA-ED, 0.942 for GATCN, 0.937 for GRU, and 0.927 for ARIMA. The 0.001 difference from the closest baselines indicates that the advantage in this illustrative period was modest, although TSLCN provided the closest overall fit across the displayed lane–class series.

Table 8 reports model size and computational cost under the implementation environment described in Section 5.1.5. TSLCN contained 41,791 parameters, approximately half the parameter count of STAEformer and fewer than Graph WaveNet, Multi-Lane ConvLSTM, and STA-ED. Inference over the complete test set took 1.16 s, less time than these larger models required, while GRU and GATCN completed inference faster. Training required 1301 s, less than that of Graph WaveNet and STA-ED but more than that of the remaining neural baselines. Taken together with the lowest mean errors in Table 5, these measurements show that TSLCN achieves a favorable balance between predictive accuracy and model compactness while maintaining competitive computational efficiency.

### 5.3. Ablation Study

Six ablation variants were evaluated in the 30% CAV penetration scenario using the same data partitions, training protocol, and remaining hyperparameters. As shown in Table 9, the w/o Temporal Encoder variant omitted both the shared gated causal TCN and the class-specific adapters. The w/o Class-specific Adapters variant retained the shared TCN but removed the class-specific bottleneck adapters. The w/o S-GAT, w/o L-GAT, and w/o C-Gate variants omitted the section, lane, and vehicle-class interaction components, respectively. The w/o SLC variant omitted all three structural components. Each variant was evaluated at the 5-, 10-, and 15-min horizons.

**Figure 11:**
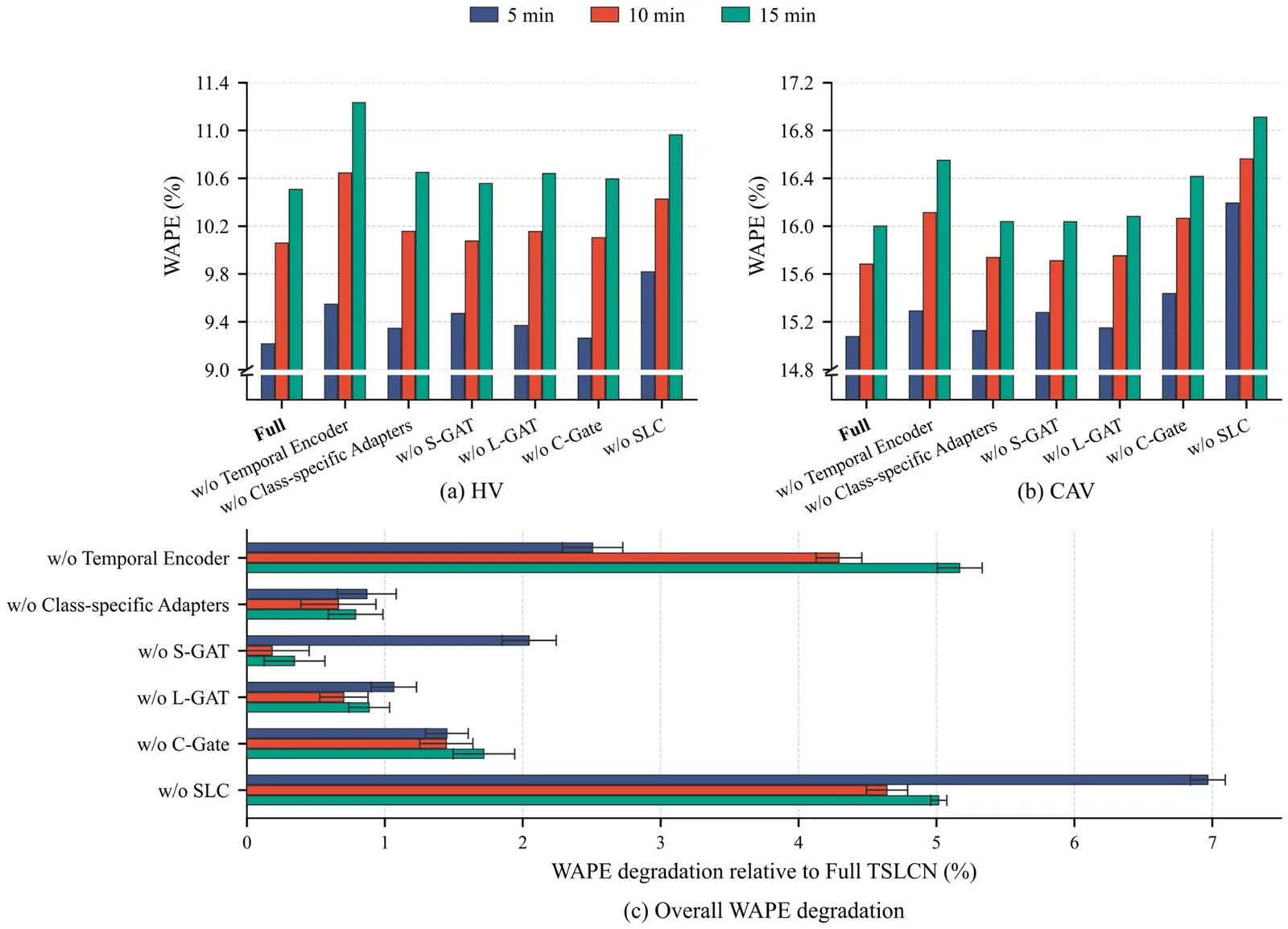
Horizon-specific ablation results.

Table 10 reports the MAE, RMSE, and WAPE pooled across all test samples, forecast horizons, monitoring sections, and lanes, together with the corresponding WAPE degradation relative to the full TSLCN. Table 11 separates the overall degradation by forecast horizon, while Figure 11 presents the class-specific WAPE and variation across the five runs. All ablated variants produced higher mean WAPE than the full model at each horizon. When the three horizons were pooled, removing the complete SLC module produced the largest overall degradation of 5.50%, followed by the temporal encoder at 4.04%. Among the individual components, C-Gate had the largest pooled effect, at 1.54%, followed by L-GAT at 0.88%, S-GAT at 0.82%, and the class-specific adapters at 0.77%.

Across all variants, WAPE increased with the forecast horizon for both vehicle classes. Removing the shared gated causal TCN together with the class-specific adapters increased overall WAPE by 2.51%, 4.29%, and 5.17% at 5, 10, and 15 min, respectively. When the shared TCN was retained, adapter removal produced increases of 0.87%, 0.66%, and 0.79%. Comparing the two temporal variants shows that, when the class-specific adapters were absent, retaining the shared TCN became increasingly beneficial as the forecast horizon lengthened. At 5 min, the adapter- removal variant produced the smallest degradation among all ablations. The relatively small effect of adapter removal suggests that the shared TCN captured much of the temporal structure common to HV and CAV flows, while the adapters provided additional class-specific refinement. The pooled adapter effect was greater for HV than for CAV, at 1.24% and 0.31%, respectively. This difference may reflect the greater temporal variability associated with heterogeneous human driving and lane-changing behavior, while the comparatively regular CAV patterns may have been represented more effectively by the shared temporal features.

The three SLC components displayed distinct horizon-dependent patterns. Removing S-GAT increased overall WAPE by 2.05%, 0.18%, and 0.35% across the three horizons. Its contribution was concentrated at 5 min, when traffic states across the monitoring sections provided more immediate information about longitudinal propagation. Removing L-GAT produced smaller but persistent increases of 1.07%, 0.70%, and 0.89%, consistent with the continuing influence of lane changes and ramp-related flow redistribution. C-Gate removal increased WAPE by 1.45%, 1.45%, and 1.72%, with a larger pooled effect on CAV than on HV (2.48% versus 0.61%). This class difference is consistent with the directional design of C-Gate, which regulates the information received by HV and CAV separately. At 30% CAV penetration, HVs formed the larger share of the local traffic state and may therefore have provided a more informative reference for the CAV representation. Among the individual SLC components, S-GAT had the largest effect at 5 min but the smallest effect at 10 and 15 min, whereas C-Gate became the most influential component at the two longer horizons.

The complete SLC ablation increased overall WAPE by 6.97%, 4.64%, and 5.02% at 5, 10, and 15 min, respectively, exceeding the effect of removing any individual SLC component at each horizon. Its pooled degradation reached 4.79% for HV and 6.22% for CAV. Removing the complete SLC module produced a larger degradation for CAVs than for HVs. In the full model, section and lane information is passed sequentially to the vehicle-class gate; removing SLC therefore eliminates both the spatial context and the subsequent exchange between HV and CAV representations.

### 5.4. Robustness and Generalization

#### 5.4.1. Performance Under Different CAV Penetration Rates

**Figure 12:**
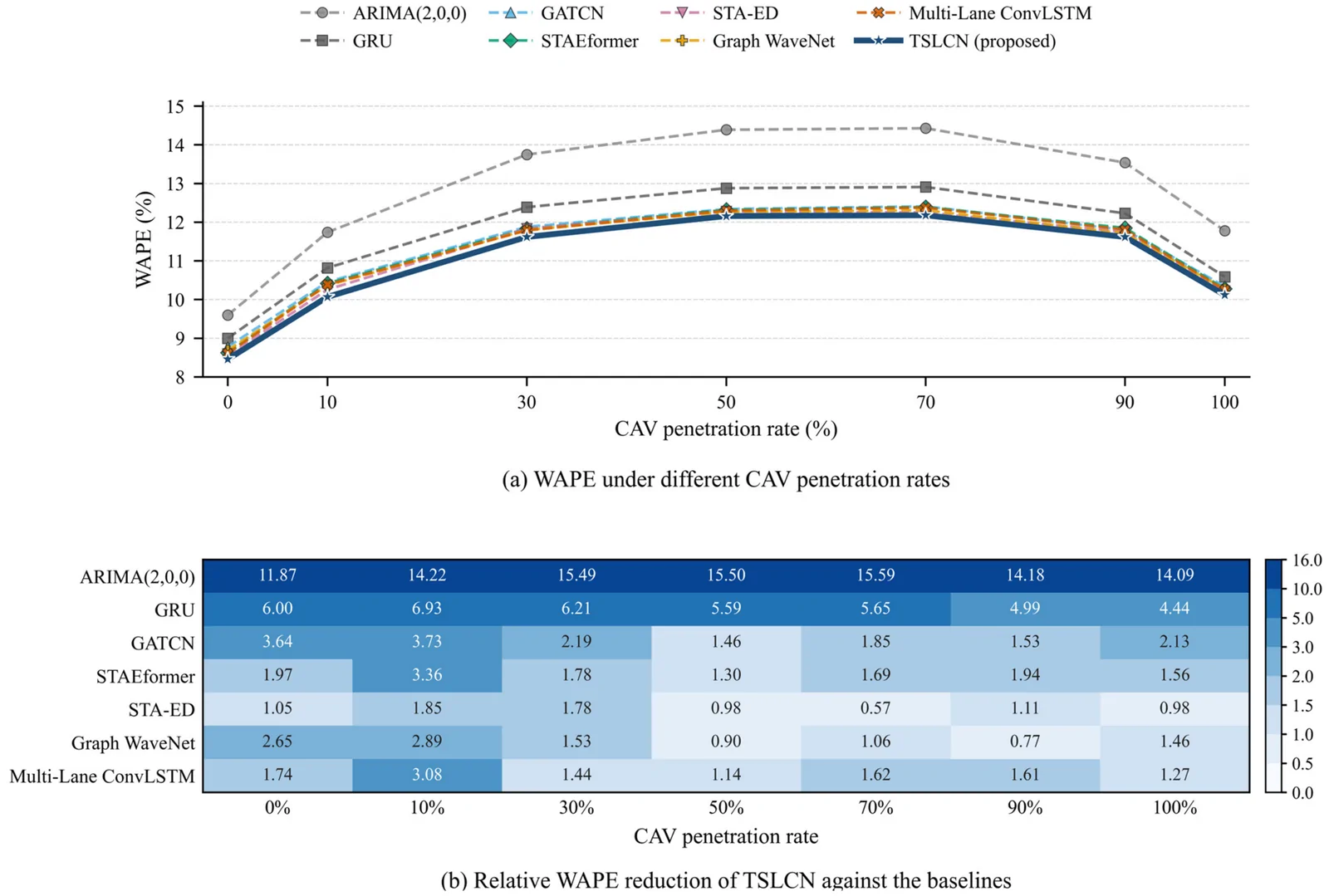
Forecasting performance under different CAV penetration rates.

To examine how traffic composition affects forecasting performance, the models were evaluated at CAV penetration rates of 0%, 10%, 30%, 50%, 70%, 90%, and 100%. Table 12 reports the overall WAPE under each setting, while Figure 12a shows the variation in prediction error and Figure 12b presents the relative WAPE reduction achieved by TSLCN against each baseline. Overall WAPE was calculated by pooling the absolute errors and observed flows over all active section–lane–class targets. At 0% penetration, only the HV targets and the HV adapter were active. At 100%, only the CAV targets and the CAV adapter were active. In both cases, the inactive class was excluded from the loss and evaluation, and the corresponding cross-class messages in C-Gate were masked. The temporal encoder, S-GAT, L-GAT, and the adapter associated with the active vehicle class remained unchanged.

As shown in Table 12 and Figure 12a, WAPE generally increased from the single-class endpoints toward the mid-range penetration levels, reaching its highest values around 50–70% penetration. For TSLCN, WAPE increased from 8.46% at 0% to 12.16% and 12.18% at 50% and 70%, respectively, before decreasing to 10.12% at 100%. This variation reflects both the heterogeneous traffic dynamics and the composition of the pooled error measure. When both vehicle classes account for substantial shares of the traffic stream, their different following and gap-acceptance characteristics introduce greater variation in flow propagation and lane-changing redistribution across sections and lanes. This heterogeneity increases the difficulty of predicting class-specific flows and contributes to the higher WAPE observed at intermediate penetration levels. Closer to the endpoints, one vehicle class becomes less prevalent, but prediction errors represent a larger proportion of its lower flow volume. At 10% penetration, TSLCN obtained WAPE values of 8.35% for HVs and 25.59% for CAVs. CAVs accounted for 9.98% of the observed flow, and their errors increased the pooled WAPE to 10.07%. At 90% penetration, the corresponding WAPE values were 25.09% for HVs and 10.13% for CAVs, resulting in an overall WAPE of 11.62%. At 0% and 100%, only one vehicle class remains in the traffic stream and evaluation, and the flow series are generated under a single vehicle-behavior regime. At the two endpoints, WAPE was determined solely by the active vehicle class and was lower than that observed at the intermediate penetration levels.

The two endpoints nevertheless differed in forecasting difficulty. Their mean lane-level flows were similar, at approximately 97.50 vehicles per 5 min in the all-HV scenario and 97.60 vehicles per 5 min in the all-CAV scenario. The all-CAV series had a standard deviation of approximately 46.50 vehicles per 5 min and a 95th percentile of 180, compared with 43.50 and 152 for the all-HV series. The corresponding TSLCN MAE values were approximately 8.25 and 9.88 vehicles per 5 min, respectively. Under the applied SUMO settings, the shorter CAV headway can support higher short-term discharge flows, which is consistent with the broader upper tail observed in the all-CAV series. The larger absolute deviations associated with these peaks account for the higher WAPE at 100% penetration despite the similar mean flow at the two endpoints.

TSLCN obtained the lowest WAPE at all seven penetration levels. Its reduction relative to the strongest baseline ranged from 0.57% to 1.85%, with the largest difference at 10% penetration and the smallest at 70%, as shown in Figure 12b. At 10% penetration, TSLCN reduced the errors of both vehicle classes, and the improvement in HV prediction had a greater influence on the pooled WAPE as HVs accounted for most of the observed flow. At 70% penetration, CAVs formed the larger share of traffic, while the leading spatial models produced similar errors for CAV flows, leaving a narrower difference in the overall WAPE. At the two endpoints, the branch associated with the absent class, including its adapter and output head, was masked and excluded from the loss calculation. C-Gate was bypassed, whereas the adapter of the active class remained in the forecasting pathway. TSLCN reduced WAPE by 1.05% relative to STA-ED at 0% penetration and by 0.98% at 100%, using the shared temporal encoder, the active-class adapter, and the section- and lane-level interactions.

#### 5.4.2. Sensitivity to Simulation Parameters

The sensitivity analysis was conducted at 30% CAV penetration, consistent with the main experimental setting. The desired time headway *τ* specifies the longitudinal spacing maintained by CAVs, while actionStepLength determines the interval between successive control updates. For lane-changing behavior, lcAssertive controls the accepted gap and lcStrategic regulates route-related lane-change planning. Each parameter was varied individually over three selected values, including the default, with traffic demand, freeway geometry, penetration rate, and the remaining simulation settings held constant. The models were retrained for each setting using the same chronological data split and optimization configuration. Table 13 reports the results for TSLCN, Multi-Lane ConvLSTM, and Graph WaveNet. The two reference models produced the lowest baseline WAPE values in the 30% penetration experiment and represent convolutional recurrent and adaptive graph-based spatial modeling.

Changes in the longitudinal behavior parameters produced clear differences in forecasting accuracy. Reducing *τ* from 0.6 to 0.4 s lowered the TSLCN WAPE from 11.62% to 11.35%, while increasing it to 0.8 s raised WAPE to 11.99%. A longer time headway increases the road space occupied by each CAV and lowers the discharge capacity available under a given demand. The resulting changes in queue formation and disturbance propagation increase short-term variability in lane-level flows and make their evolution more difficult to predict. A larger response was observed for actionStepLength, with WAPE increasing to 11.87% at 0.3 s and 12.20% at 0.5 s. Extending the interval between vehicle decisions reduces the frequency at which acceleration and lane-changing actions are recomputed, allowing changes in spacing and speed to persist longer before the next update.

The lane-changing parameters had more moderate effects. Reducing lcAssertive from 0.7 to 0.5 lowered WAPE to 11.46%, whereas increasing it to 1.0 raised WAPE to 11.97%. The acceptance of smaller gaps at higher values can generate sharper braking and acceleration responses in the target lane, increasing short-term fluctuations in lane-level flow. The response to lcStrategic was non-monotonic, with WAPE values of 11.86%, 11.62%, and 11.68% at 0.25, 0.5, and 1.0, respectively. Lower values tend to postpone route-related lane changes toward the diverging area, whereas higher values initiate more of these maneuvers farther upstream. Changes in their spatial distribution can therefore alter the lane-level flow patterns observed at successive monitoring sections. The lowest error occurred at the default setting, while deviations in either direction produced relatively small increases in WAPE.

Measured by the maximum absolute departure from the default WAPE, actionStepLength produced the largest change in TSLCN, at 0.58 percentage points, followed by *τ* at 0.37, lcAssertive at 0.35, and lcStrategic at 0.24. Multi-Lane ConvLSTM showed a similar response to actionStepLength, with a maximum change of 0.55 percentage points, while Graph WaveNet reached its maximum change of 0.38 percentage points under both *τ* and actionStepLength. TSLCN recorded the lowest WAPE in seven of the nine unique configurations. Graph WaveNet returned marginally lower values at actionStepLength=0.5 s and lcStrategic=1.0, with differences of 0.02 and 0.01 percentage points, respectively. Across the full set of configurations, TSLCN WAPE ranged from 11.35% to 12.20%, while its relative position among the three models remained stable.

#### 5.4.3. Evaluation on an Alternative Freeway

**Figure 13:**
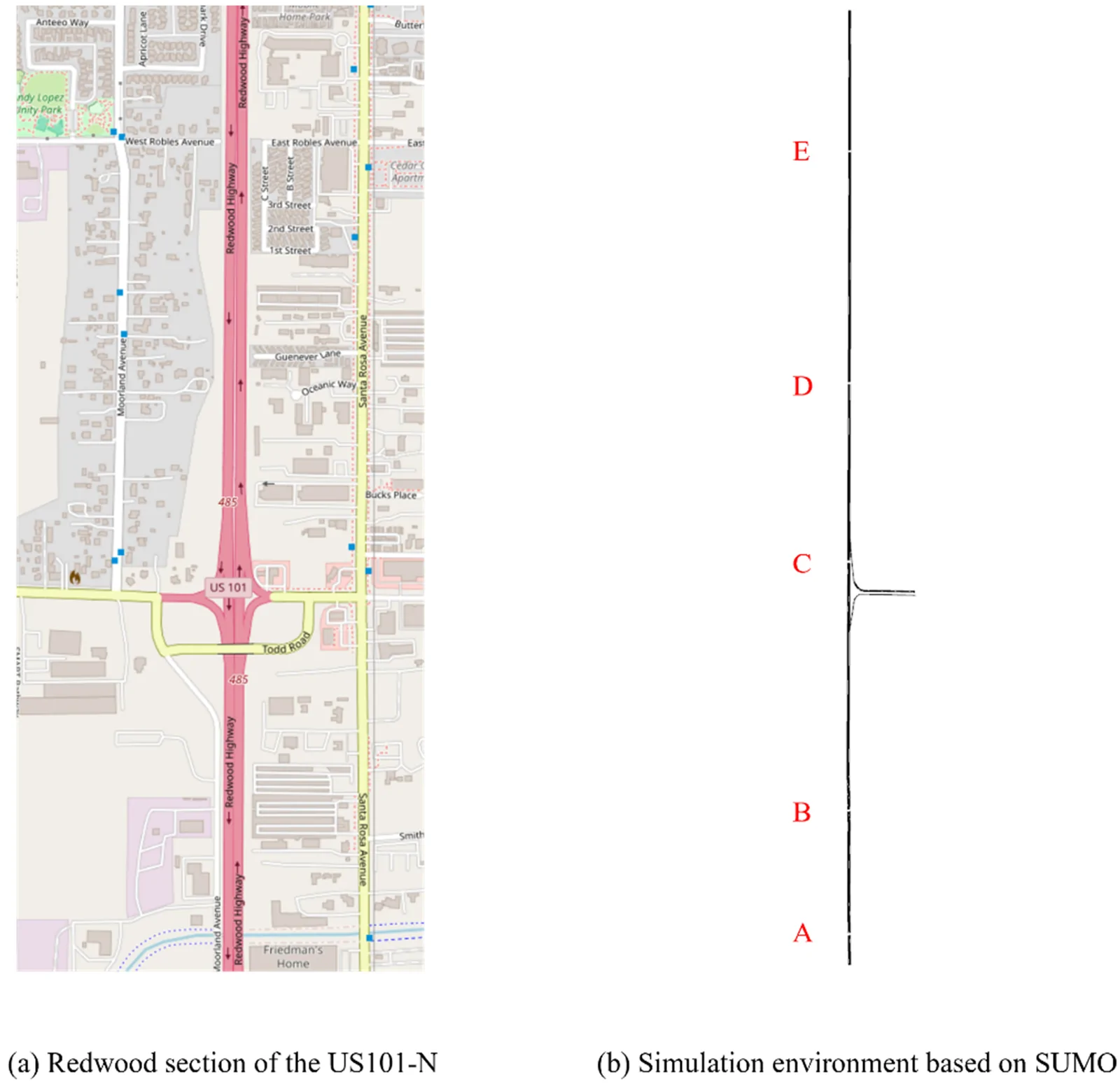
Study corridor of alternative freeway and SUMO simulation environment.

An additional evaluation was conducted on a 3.1-km northbound section of US 101 in Redwood City to examine forecasting performance under a different freeway configuration. Figure 13 shows the study corridor and its SUMO representation. As summarized in Table 14, the alternative corridor contains three mainline lanes, one on-ramp, and one off-ramp. The original 2.2-km corridor contains four lanes, one on-ramp, and two off-ramps. Both corridors use five monitoring sections and a 5-min flow resolution. In the mixed-traffic evaluation, reducing the number of lanes from four to three decreased the number of section–lane–class targets from 40 to 30 and reduced the number of lateral relationships represented by the models.

The evaluation used field-observed lane-level traffic and simulated mixed traffic on the alternative corridor. Field-observed 5-min lane flows were obtained from PeMS for 1–31 August 2018. As vehicle-class labels were unavailable, the field observations were used for single-class lane-flow evaluation, with all observed flows assigned to the HV channel. Only the HV targets and HV adapter were active, while the CAV branch was excluded and cross-class exchange through C-Gate was bypassed. A separate mixed-traffic dataset was generated in SUMO on the same freeway geometry at 30% CAV penetration using the vehicle parameters and simulation settings described in Section 5.1.2. Both vehicle-class branches and all TSLCN components were active in this experiment. The models were retrained separately on the two datasets using the data partition, historical input window, forecast horizons, and optimization procedure adopted in the main experiment.

Table 15 summarizes the forecasting results on the alternative corridor. For the field-observed traffic, TSLCN achieved an MAE of 4.45, an RMSE of 5.87, and a WAPE of 5.47%. Multi-Lane ConvLSTM was the strongest baseline, with corresponding values of 4.53, 6.11, and 5.57%. TSLCN reduced these errors by 1.77%, 3.93%, and 1.80%, respectively. For the simulated mixed traffic, TSLCN obtained an MAE of 3.28, an RMSE of 4.48, and a WAPE of 8.05%. Graph WaveNet provided the strongest baseline results of 3.35, 4.63, and 8.22%. The corresponding reductions were 2.09%, 3.24%, and 2.07%.

Prediction errors were lower on the alternative corridor than in the original 30% CAV experiment. The lower traffic volume reduced the scale of the prediction targets and was associated with smaller MAE and RMSE values. The straighter alignment, lower ramp density, and clearer daily flow patterns also corresponded to more regular spatial and temporal variation. Within the alternative corridor, the field observations produced higher MAE and RMSE but lower WAPE than the simulated mixed traffic. The field evaluation involved one lane-flow target for each section–lane unit, whereas the mixed-traffic evaluation predicted HV and CAV flows separately. The smaller class-specific targets partly explain the lower MAE and RMSE. For WAPE, the absolute errors of the HV and CAV forecasts were summed and normalized by their combined observed flow. Consequently, overprediction of one vehicle class and underprediction of the other both increased WAPE, even when the two errors partially cancelled after flow aggregation. The separate accumulation of class-specific errors, together with variation in vehicle arrivals and lane-changing movements, was associated with the higher WAPE of the mixed-traffic forecasts.

The ordering of the baseline models changed with the traffic setting. Multi-Lane ConvLSTM performed best among the baselines for the field observations, consistent with its representation of local continuity in the regular lane-flow patterns. Graph WaveNet became the strongest baseline for the simulated mixed traffic, where its adaptive directed connections represented spatial dependencies under the alternative freeway configuration. TSLCN achieved the lowest MAE, RMSE, and WAPE under both settings. For the simulated mixed traffic, its reductions over the strongest baseline were 2.09% for MAE, 3.24% for RMSE, and 2.07% for WAPE, compared with 1.45%, 1.18%, and 1.45% in the main experiment. The relative performance margin increased most markedly for RMSE. The changes in baseline ranking were associated with the freeway configuration and the organization of the prediction targets.

## 6. Conclusions

This study formulated lane-level mixed traffic prediction as joint forecasting of HV and CAV flows across freeway sections and lanes. The proposed TSLCN combines shared temporal encoding and class-specific adaptation with longitudinal, lateral, and cross-class interaction modeling. The main experiments used mixed traffic simulated on the original freeway with demand derived from field observations. Additional experiments examined robustness to traffic composition and simulation parameters, as well as adaptability to another freeway configuration. The principal findings are summarized as follows.

(1) In the main 30% CAV scenario, TSLCN achieved the lowest aggregate MAE, RMSE, and WAPE. Its horizon-specific MAE differences from the strongest baselines remained statistically significant after Holm correction. TSLCN also recorded the lowest mean error in 68 of the 72 lane–class–horizon–metric comparisons. GATCN produced slightly lower errors in the other four cases, all involving longer-horizon CAV forecasts in Lane 3. Fine-grained analysis showed that errors generally increased with forecast horizon, while CAV flows and the ramp-connected outer lane had higher relative errors. TSLCN achieved this performance with fewer parameters and shorter inference time than the four larger spatial baselines.

(2) The ablation analysis showed that temporal encoding, class-specific adaptation, and the SLC interactions contributed complementary information. Every ablated variant increased WAPE at all three forecast horizons. Removing the complete SLC module produced the largest pooled degradation of 5.50%, followed by the 4.04% increase caused by removing the temporal encoder. The influence of each component varied with forecast horizon and vehicle class, reflecting the different temporal and interaction patterns represented within TSLCN.

(3) TSLCN performed consistently across different traffic compositions, simulation settings, and freeway configurations. It achieved the lowest WAPE at all seven CAV penetration levels, including the 0% and 100% endpoints, where only one vehicle-class branch was active. TSLCN also ranked first in seven of the nine parameter-sensitivity settings, while Graph WaveNet recorded slightly lower WAPE in the remaining two. Cross-corridor adaptability was further examined on an alternative freeway with a different lane number and ramp configuration. After retraining, TSLCN achieved the lowest MAE, RMSE, and WAPE for both field-observed lane flows and simulated mixed traffic. These results indicate that its section–lane representation remained effective under a different freeway topology.

Future work will extend class-specific validation to field observations as suitable mixed-traffic datasets become available and assess transfer across additional freeway corridors with limited retraining. It will also examine interactions among traffic demand, car-following behavior, and lane-changing behavior. A multivariate probabilistic extension incorporating speed and occupancy could broaden traffic-state representation and quantify forecast uncertainty.

## Figures and Tables

**Table 1:** Comparison of representative lane-level traffic prediction formulations.

Model	Forecast Target	Vehicle Representation	Spatial and Cross-Series Modeling	Prediction Scope
FDL [27]	Speed at a selected lane section	Aggregate vehicle classes	Entropy-based grey relational analysis for input selection, followed by LSTM–GRU temporal modeling	One target lane section
ST-AFN [28]	Flow at a selected lane	Aggregate vehicle classes	Speed-feature processing, spatial attention over candidate lane series, and temporal-attention decoding	One target-lane flow sequence
MDL [29]	Speeds at multiple section–lane units	Aggregate vehicle classes	MIC-based input selection; section–lane arrays processed by ConvLSTM and convolutional layers	Multiple section–lane speeds forecast jointly
GraphMLP [6]	One speed or flow variable at each lane-segment node	Aggregate vehicle classes	Dynamic lane-segment graph learning, independent temporal MLP modeling, and gated spatiotemporal fusion	All lane-segment nodes forecast jointly for one traffic variable
STA-ED [35]	Flow of one vehicle class at a target lane section	Small and medium-sized passenger cars; small and medium-sized trucks; large buses and trucks	Spatial attention over lane–vehicle-type sequences and temporal attention within an LSTM encoder–decoder	One lane–class flow sequence
DATCN [7]	HV or CAV flow at a selected section–lane unit	HV and CAV	Target-specific DTW input selection, feature attention, and TCN temporal modeling	One section–lane–class flow sequence

**Table 2:** Simulation parameters of different types of vehicles.

Parameter	HV	CAV
carFollowModel	IDM	CACC
tau	1.5 s	0.6 s
minGap	2 m	1 m
actionStepLength	0.5 s	0.1 s
lcAssertive	3.0	0.7
lcSpeedGain	2.5	0.5
lcStrategic	1.0	0.5
lcCooperative	0.5	1.0
speedFactor	normc (1, 0.1, 0.2, 2)	1
laneChangeModel	LC2013	
length	5 m	

**Table 3:** Model-specific configurations of the baseline methods.

Baseline	Core Structure	Model-Specific Settings
ARIMA	Independent autoregression for each flow series	Order (2,0,0)
GRU	Shared univariate GRU	One layer; hidden dimension of 32
GATCN	Gated temporal convolutions and graph attention over adjacent-section and adjacent-lane edges	Two temporal layers; kernel size of 3; dilation of 1; one attention head; hidden dimension of 32
Graph WaveNet	Dilated temporal convolutions and diffusion convolution over forward, backward, and adaptive graph supports	Two blocks with four layers each; kernel size of 2; dilations of 1, 2, 4, and 8; diffusion order of 2; adaptive embedding dimension of 10; residual/skip/end channels: 32/64/128
STAEformer	Adaptive embedding followed by temporal and spatial self-attention	Input and adaptive embedding dimensions: 24/40; model dimension of 64; four attention heads; one temporal and one spatial layer; feed-forward dimension of 128
Multi-Lane ConvLSTM	Stacked ConvLSTM over the section–lane grid	Two layers; 32 hidden channels; 3 × 3 kernels
STA-ED	Target-specific input attention and temporal-attention LSTM encoder–decoder	64 hidden and attention dimensions; independent parameters for each prediction target

**Table 4:** Common training settings for the neural models.

Parameter	Setting
Optimizer	AdamW
Batch size	64
Initial learning rate	1 × 10^−3^
Learning-rate scheduler	ReduceLROnPlateau; factor = 0.5; patience = 5 epochs
Weight decay	1 × 10^−4^
Gradient clipping norm	5
Early-stopping patience	15 epochs
Maximum number of epochs	150
Random seeds	42, 52, 62, 72, and 82

**Table 5:** Overall performance of the compared models.

Model	MAE	RMSE	WAPE (%)
ARIMA (2,0,0)	6.71	8.93	13.75
GRU	6.04 ± 0.009	8.20 ± 0.006	12.39 ± 0.017
GATCN	5.79 ± 0.006	7.92 ± 0.010	11.88 ± 0.013
STAEformer	5.77 ± 0.016	7.86 ± 0.013	11.83 ± 0.033
STA-ED	5.77 ± 0.009	7.91 ± 0.011	11.83 ± 0.019
Graph WaveNet	5.76 ± 0.028	7.83 ± 0.015	11.80 ± 0.057
Multi-Lane ConvLSTM	5.75 ± 0.012	7.86 ± 0.022	11.79 ± 0.026
**TSLCN (proposed)**	**5.67 ± 0.006**	**7.74 ± 0.009**	**11.62 ± 0.012**

Note: Results for the neural models are reported as the mean ± SD over five random seeds. ARIMA is deterministic under the fixed data and model order.

**Table 6:** Horizon-specific comparison of TSLCN with the strongest baseline.

Horizon	Strongest Baseline	Baseline MAE	TSLCN MAE	ΔMAE (95% CI)	Reduction	DM Statistic	Holm *p*
5 min	STAEformer	5.44 ± 0.019	**5.35 ± 0.005**	0.093 [0.064, 0.121]	1.70%	6.363	<0.001
10 min	Graph WaveNet	5.79 ± 0.033	**5.73 ± 0.008**	0.064 [0.033, 0.096]	1.11%	3.959	<0.001
15 min	Multi-Lane ConvLSTM	5.99 ± 0.013	**5.93 ± 0.009**	0.062 [0.018, 0.107]	1.04%	2.731	0.006

**Table 7:** Lane-specific multi-step prediction performance of the compared models for HVs and CAVs at 30% CAV penetration.

(**a**) Lane 1										
**Model**	**V-Class**	**5 min**			**10 min**			**15 min**		
		**MAE**	**RMSE**	**WAPE**	**MAE**	**RMSE**	**WAPE**	**MAE**	**RMSE**	**WAPE**
ARIMA (2,0,0)	HV	7.50	10.09	8.79	8.49	11.57	9.94	9.12	12.37	10.69
	CAV	5.73	7.38	17.07	6.09	7.76	18.14	6.46	8.18	19.25
GRU	HV	7.22	9.78	8.46	7.85	10.72	9.20	8.36	11.38	9.79
	CAV	5.17	6.79	15.40	5.26	6.95	15.69	5.45	7.22	16.26
GATCN	HV	6.85	9.31	8.02	7.46	10.47	8.74	7.93	11.18	9.29
	CAV	4.91	6.41	14.63	5.08	6.70	15.15	5.23	6.93	15.60
STAEformer	HV	6.82	9.19	7.99	7.55	10.45	8.85	8.02	11.13	9.40
	CAV	4.88	6.37	14.54	5.12	6.73	15.25	5.22	6.91	15.57
STA-ED	HV	6.91	9.44	8.09	7.49	10.47	8.78	7.91	11.06	9.27
	CAV	4.89	6.45	14.59	5.06	6.69	15.07	5.18	6.89	15.46
Graph WaveNet	HV	6.78	9.09	7.94	7.39	10.29	8.66	7.88	10.95	9.23
	CAV	4.90	6.38	14.60	5.10	6.68	15.19	5.21	6.92	15.55
Multi-Lane ConvLSTM	HV	6.85	9.25	8.03	7.43	10.37	8.71	7.82	11.01	9.17
	CAV	4.89	6.38	14.58	5.07	6.69	15.12	5.21	6.90	15.53
**TSLCN (proposed)**	HV	6.56 *	8.91 *	7.69 *	7.24 *	10.10 *	8.48 *	7.63 *	10.73 *	8.94 *
	CAV	4.79 *	6.28 *	14.28 *	5.04 *	6.65 *	15.03 *	5.14 *	6.83 *	15.33 *
(**b**) Lane 2										
**Model**	**V-Class**	**5 min**			**10 min**			**15 min**		
		**MAE**	**RMSE**	**WAPE**	**MAE**	**RMSE**	**WAPE**	**MAE**	**RMSE**	**WAPE**
ARIMA (2,0,0)	HV	7.48	9.68	9.87	8.22	10.87	10.85	8.70	11.55	11.49
	CAV	5.63	7.23	16.93	6.02	7.65	18.09	6.17	7.73	18.53
GRU	HV	7.05	9.25	9.31	7.49	9.99	9.89	7.90	10.54	10.42
	CAV	5.12	6.61	15.37	5.25	6.81	15.78	5.39	7.00	16.21
GATCN	HV	6.75	8.81	8.91	7.25	9.75	9.57	7.62	10.32	10.05
	CAV	4.80	6.19	14.42	4.98	6.46	14.97	5.12	6.65	15.37
STAEformer	HV	6.69	8.70	8.83	7.29	9.76	9.62	7.65	10.33	10.10
	CAV	4.80	6.18	14.42	5.00	6.49	15.03	5.11	6.67	15.36
STA-ED	HV	6.76	8.90	8.92	7.30	9.83	9.64	7.61	10.30	10.04
	CAV	4.83	6.24	14.51	4.99	6.48	15.00	5.11	6.65	15.34
Graph WaveNet	HV	6.64	8.61	8.76	7.20	9.67	9.50	7.56	10.23	9.98
	CAV	4.80	6.19	14.42	4.97	6.44	14.94	5.11	6.67	15.34
Multi-Lane ConvLSTM	HV	6.71	8.74	8.86	7.26	9.74	9.58	7.55	10.23	9.96
	CAV	4.81	6.19	14.46	4.97	6.45	14.94	5.10	6.65	15.33
**TSLCN (proposed)**	HV	6.55 *	8.55 *	8.65 *	7.15 *	9.58 *	9.44 *	7.46 *	10.10 *	9.85 *
	CAV	4.72 *	6.10 *	14.19 *	4.92 *	6.41 *	14.79 *	5.06 *	6.61 *	15.21 *
(**c**) Lane 3										
**Model**	**V-Class**	**5 min**			**10 min**			**15 min**		
		**MAE**	**RMSE**	**WAPE**	**MAE**	**RMSE**	**WAPE**	**MAE**	**RMSE**	**WAPE**
ARIMA (2,0,0)	HV	7.27	9.42	11.53	8.08	10.60	12.80	8.46	11.07	13.41
	CAV	5.31	6.89	18.61	5.67	7.34	19.87	5.76	7.25	20.17
GRU	HV	6.77	8.95	10.73	7.23	9.66	11.46	7.57	10.11	12.00
	CAV	4.78	6.22	16.76	4.91	6.40	17.21	4.98	6.48	17.45
GATCN	HV	6.58	8.62	10.43	7.08	9.49	11.23	7.44	9.98	11.78
	CAV	4.46	5.80	15.62	4.58 *	6.02 *	16.06 *	4.67	6.13 *	16.37
STAEformer	HV	6.34	8.31	10.05	7.04	9.39	11.15	7.38	9.93	11.70
	CAV	4.43	5.76	15.51	4.62	6.05	16.18	4.70	6.15	16.47
STA-ED	HV	6.44	8.55	10.21	7.00	9.45	11.10	7.33	9.92	11.61
	CAV	4.46	5.82	15.62	4.63	6.09	16.22	4.71	6.18	16.49
Graph WaveNet	HV	6.43	8.39	10.20	7.02	9.37	11.13	7.40	9.92	11.73
	CAV	4.46	5.81	15.64	4.63	6.06	16.22	4.70	6.16	16.46
Multi-Lane ConvLSTM	HV	6.43	8.46	10.20	7.04	9.45	11.16	7.35	9.91	11.64
	CAV	4.45	5.78	15.59	4.61	6.06	16.17	4.69	6.16	16.45
**TSLCN (proposed)**	HV	6.29 *	8.29 *	9.97 *	6.96 *	9.34 *	11.02 *	7.29 *	9.83 *	11.55 *
	CAV	4.41 *	5.74 *	15.45 *	4.59	6.03	16.08	4.67 *	6.14	16.36 *
(**d**) Lane 4										
**Model**	**V-Class**	**5 min**			**10 min**			**15 min**		
		**MAE**	**RMSE**	**WAPE**	**MAE**	**RMSE**	**WAPE**	**MAE**	**RMSE**	**WAPE**
ARIMA (2,0,0)	HV	6.71	8.69	13.72	7.16	9.27	14.62	7.46	9.63	15.24
	CAV	4.35	5.53	20.10	4.53	5.75	20.95	4.56	5.78	21.09
GRU	HV	6.22	8.16	12.72	6.42	8.46	13.13	6.62	8.69	13.53
	CAV	3.98	5.07	18.37	4.02	5.14	18.57	4.03	5.16	18.65
GATCN	HV	6.06	7.88	12.38	6.27	8.25	12.81	6.48	8.50	13.23
	CAV	3.78	4.84	17.45	3.84	4.93	17.75	3.87	4.97	17.89
STAEformer	HV	5.85	7.64	11.95	6.18	8.14	12.62	6.33	8.38	12.94
	CAV	3.75	4.79	17.32	3.85	4.92	17.80	3.89	4.98	17.98
STA-ED	HV	5.89	7.72	12.03	6.18	8.19	12.64	6.34	8.38	12.95
	CAV	3.78	4.84	17.47	3.84	4.92	17.73	3.87	4.95	17.88
Graph WaveNet	HV	5.91	7.73	12.08	6.20	8.18	12.67	6.40	8.43	13.08
	CAV	3.76	4.82	17.37	3.84	4.92	17.72	3.86	4.95	17.85
Multi-Lane ConvLSTM	HV	5.86	7.65	11.97	6.16	8.14	12.60	6.33	8.39	12.94
	CAV	3.75	4.80	17.33	3.81	4.90	17.62	3.86	4.95	17.82
**TSLCN (proposed)**	HV	5.76 *	7.53 *	11.77 *	6.13 *	8.12 *	12.52 *	6.31 *	8.36 *	12.90 *
	CAV	3.72 *	4.75 *	17.19 *	3.80 *	4.87 *	17.55 *	3.85 *	4.93 *	17.78 *

Note: Each entry is calculated by pooling predictions from the five monitoring sections for the specified lane, vehicle class, and forecast horizon. Entries are five-run means rounded to two decimal places. * indicates the lowest displayed mean in each comparison.

**Table 8:** Model complexity and computational efficiency of the compared models.

Model	Parameters	Best Epoch	Training Time (s)	Inference Time (s)
ARIMA (2,0,0)	N/A	—	—	0.42
GRU	6563	79	747	0.48
GATCN	15,014	70	524	0.43
STAEformer	84,782	18	888	1.37
STA-ED	2,652,280	26	2882	3.18
Graph WaveNet	126,326	17	1650	3.58
Multi-Lane ConvLSTM	150,310	10	458	1.25
TSLCN (proposed)	41,791	56	1301	1.16

Note: Parameter counts are fixed model properties, whereas the reported training and inference times are medians over five runs. “—” indicates that the metric is not defined for statistical models without iterative weight-updating procedures.

**Table 9:** Configurations of the TSLCN ablation study.

Model	Temporal Encoder	Class-Specific Adapters	S-GAT	L-GAT	C-Gate
TSLCN (Full)	√	√	√	√	√
TSLCN w/o Temporal Encoder	—	—	√	√	√
TSLCN w/o Class-specific Adapters	√	—	√	√	√
TSLCN w/o S-GAT	√	√	—	√	√
TSLCN w/o L-GAT	√	√	√	—	√
TSLCN w/o C-Gate	√	√	√	√	—
TSLCN w/o SLC	√	√	—	—	—

Note: “√” indicates that the corresponding component is retained, whereas “—“ indicates that it is removed.

**Table 10:** Pooled prediction errors across the three forecast horizons in the ablation study.

Model	HV				CAV				Overall Deg. (%)
	MAE	RMSE	WAPE (%)	Deg. (%)	MAE	RMSE	WAPE (%)	Deg. (%)	
**Full**	**6.78**	**9.17**	**9.93**	**—**	**4.56**	**5.99**	**15.59**	**—**	**—**
w/o Temporal Encoder	7.15	9.66	10.47	5.52	4.68	6.12	15.98	2.56	4.04
w/o Class-specific Adapters	6.86	9.27	10.05	1.24	4.57	6.01	15.63	0.31	0.77
w/o S-GAT	6.85	9.27	10.03	1.08	4.58	6.02	15.67	0.57	0.82
w/o L-GAT	6.86	9.27	10.05	1.28	4.58	6.01	15.66	0.48	0.88
w/o C-Gate	6.82	9.23	9.99	0.61	4.67	6.14	15.97	2.48	1.54
w/o SLC	7.10	9.57	10.40	4.79	4.84	6.35	16.56	6.22	5.50

Note: MAE, RMSE, and WAPE are calculated by pooling predictions over all test samples, three forecast horizons (5, 10, and 15 min), five monitoring sections, and four lanes. WAPE degradation (Deg.) denotes the percentage increase in WAPE of an ablated variant relative to the Full TSLCN for the corresponding vehicle class. Overall Deg. is the mean of the corresponding HV and CAV values. All degradation values are calculated from unrounded WAPE values.

**Table 11:** Horizon-specific overall WAPE degradation relative to the Full TSLCN.

Ablated Variant	5 min (%)	10 min (%)	15 min (%)
w/o Temporal Encoder	2.51	4.29	5.17
w/o Class-specific Adapters	0.87	0.66	0.79
w/o S-GAT	2.05	0.18	0.35
w/o L-GAT	1.07	0.70	0.89
w/o C-Gate	1.45	1.45	1.72
w/o SLC	6.97	4.64	5.02

Note: For each forecast horizon, WAPE degradation was calculated separately for HV and CAV, and the two class-specific values were averaged to obtain the overall degradation.

**Table 12:** WAPE of the forecasting models under different CAV penetration rates.

Model	0%	10%	30%	50%	70%	90%	100%
ARIMA (2,0,0)	9.60	11.74	13.75	14.39	14.43	13.54	11.78
GRU	9.00	10.82	12.39	12.88	12.91	12.23	10.59
GATCN	8.78	10.46	11.88	12.34	12.41	11.80	10.34
STAEformer	8.63	10.42	11.83	12.32	12.39	11.85	10.28
STA-ED	8.55	10.26	11.83	12.28	12.25	11.75	10.22
Graph WaveNet	8.69	10.37	11.80	12.27	12.31	11.71	10.27
Multi-Lane ConvLSTM	8.61	10.39	11.79	12.30	12.38	11.81	10.25
**TSLCN**	**8.46**	**10.07**	**11.62**	**12.16**	**12.18**	**11.62**	**10.12**

Note: The lowest value at each penetration rate is shown in bold. Overall WAPE pools absolute errors and observed flows over all active test targets, forecast horizons, monitoring sections, lanes, and vehicle classes. At 0% and 100% CAV penetration, only the HV and CAV targets, respectively, are included.

**Table 13:** Sensitivity of forecasting performance to SUMO parameter settings at 30% CAV penetration.

Parameter	Value	Multi-Lane ConvLSTM WAPE (%)	Graph WaveNet WAPE (%)	TSLCN WAPE (%)	TSLCN Change from Default (p.p.)	Better Baseline- TSLCN (p.p.)
*τ* (s)	0.4	11.52	11.46	**11.35**	−0.27	+0.11
	0.6 (default)	11.79	11.80	**11.62**	—	+0.17
	0.8	12.12	12.18	**11.99**	+0.37	+0.13
actionStepLength (s)	0.1 (default)	11.79	11.80	**11.62**	—	+0.17
	0.3	12.02	11.92	**11.87**	+0.25	+0.05
	0.5	12.34	**12.18**	12.20	+0.58	−0.02
lcAssertive	0.5	11.61	11.57	**11.46**	−0.16	+0.11
	0.7 (default)	11.79	11.80	**11.62**	—	+0.17
	1.0	12.22	12.16	**11.97**	+0.35	+0.19
lcStrategic	0.25	12.07	12.02	**11.86**	+0.24	+0.16
	0.5 (default)	11.79	11.80	**11.62**	—	+0.17
	1.0	11.76	**11.67**	11.68	+0.06	−0.01

Note: One parameter was varied at a time. p.p. denotes percentage points. TSLCN change is measured from the default-setting WAPE; positive values indicate higher error. Better baseline—TSLCN is calculated using the lower WAPE of the two reference baselines; positive values favor TSLCN. Bold values indicate the lowest WAPE in each row.

**Table 14:** Comparison of the original and alternative freeway configurations.

Configuration	Original Freeway	Alternative Freeway
Mainline lanes	4	3
Monitoring sections	5	5
Corridor length	2.2 km	3.1 km
On-ramps	1	1
Off-ramps	2	1
Flow resolution	5 min	5 min

**Table 15:** Forecasting performance on the alternative freeway for field-observed lane-level traffic and simulated mixed traffic.

(**a**) Field-observed lane-level traffic (without CAV).			
**Model**	**MAE**	**RMSE**	**WAPE (%)**
ARIMA (2,0,0)	6.26	8.72	7.69
GRU	5.42	7.27	6.65
GATCN	5.27	7.14	6.47
STA-ED	4.80	6.46	5.89
Graph WaveNet	4.64	6.24	5.70
STAEformer	4.63	6.20	5.69
Multi-Lane ConvLSTM	4.53	6.11	5.57
**TSLCN**	**4.45**	**5.87**	**5.47**
(**b**) Simulated lane-level mixed traffic at 30% CAV penetration.			
**Model**	**MAE**	**RMSE**	**WAPE (%)**
ARIMA (2,0,0)	4.32	6.11	10.60
GRU	3.84	5.35	9.42
GATCN	3.75	5.38	9.20
STA-ED	3.50	4.88	8.59
Multi-Lane ConvLSTM	3.43	4.77	8.43
STAEformer	3.38	4.65	8.29
Graph WaveNet	3.35	4.63	8.22
**TSLCN**	**3.28**	**4.48**	**8.05**

## Data Availability

The data supporting the findings of this study are available from the corresponding author upon reasonable request.
